# Relative Vaccine Effectiveness of Cell- vs Egg-Based Quadrivalent Influenza Vaccine Against Test-Confirmed Influenza Over 3 Seasons Between 2017 and 2020 in the United States

**DOI:** 10.1093/ofid/ofae175

**Published:** 2024-05-02

**Authors:** Alicia N Stein, Carrie W Mills, Ian McGovern, Kimberly W McDermott, Alex Dean, Alina N Bogdanov, Sheena G Sullivan, Mendel D M Haag

**Affiliations:** Centre for Outcomes Research and Epidemiology, CSL Seqirus, Melbourne, Australia; Real World Evidence, Veradigm, Chicago, Illinois, USA; Centre for Outcomes Research and Epidemiology, CSL Seqirus, Waltham, Massachusetts, USA; Real World Evidence, Veradigm, Chicago, Illinois, USA; Real World Evidence, Veradigm, Chicago, Illinois, USA; Real World Evidence, Veradigm, Chicago, Illinois, USA; WHO Collaborating Centre for Reference and Research on Influenza, Royal Melbourne Hospital, and Department of Infectious Diseases, University of Melbourne, at the Peter Doherty Institute of Infection and Immunity, Melbourne, Australia; Department of Epidemiology, University of California, Los Angeles, California, USA; Centre for Outcomes Research and Epidemiology, CSL Seqirus, Amsterdam, Netherlands

**Keywords:** cell-based quadrivalent influenza vaccine, egg adaptation, influenza, influenza virus mismatch, relative vaccine effectiveness

## Abstract

**Background:**

Influenza vaccine viruses grown in eggs may acquire egg-adaptive mutations that may reduce antigenic similarity between vaccine and circulating influenza viruses and decrease vaccine effectiveness. We compared cell- and egg-based quadrivalent influenza vaccines (QIVc and QIVe, respectively) for preventing test-confirmed influenza over 3 US influenza seasons (2017–2020).

**Methods:**

Using a retrospective test-negative design, we estimated the relative vaccine effectiveness (rVE) of QIVc vs QIVe among individuals aged 4 to 64 years who had an acute respiratory or febrile illness and were tested for influenza in routine outpatient care. Exposure, outcome, and covariate data were obtained from electronic health records linked to pharmacy and medical claims. Season-specific rVE was estimated by comparing the odds of testing positive for influenza among QIVc vs QIVe recipients. Models were adjusted for age, sex, geographic region, influenza test date, and additional unbalanced covariates. A doubly robust approach was used combining inverse probability of treatment weights with multivariable regression.

**Results:**

The study included 31 824, 33 388, and 34 398 patients in the 2017–2018, 2018–2019, and 2019–2020 seasons, respectively; ∼10% received QIVc and ∼90% received QIVe. QIVc demonstrated superior effectiveness vs QIVe in prevention of test-confirmed influenza: rVEs were 14.8% (95% CI, 7.0%–22.0%) in 2017–2018, 12.5% (95% CI, 4.7%–19.6%) in 2018–2019, and 10.0% (95% CI, 2.7%–16.7%) in 2019–2020.

**Conclusions:**

This study demonstrated consistently superior effectiveness of QIVc vs QIVe in preventing test-confirmed influenza over 3 seasons characterized by different circulating viruses and degrees of egg adaptation.

## Teaser

Cell-based quadrivalent influenza vaccines were superior to egg-based ones in the prevention of test-confirmed influenza among children and adults over the 2017–2018, 2018–2019, and 2019–2020 influenza seasons in the United States. These findings corroborate previously published relative effectiveness studies of cell- vs egg-based quadrivalent influenza vaccines.

Vaccines are the primary means to reduce the significant morbidity, mortality, and high-cost burden of influenza [1–4]. The majority of influenza vaccines are produced with fertilized chicken eggs. However, efficient growth of human influenza virus in eggs requires the vaccine seed viruses to adapt to avian receptors. This process, called *egg adaptation*, often leads to mutations in antigenic sites of the viral hemagglutinin (HA) protein that can alter the antigenicity of the vaccine and impair the recipient's immune response to circulating virus, thereby reducing effectiveness [5–9]. The use of propagation methods not based on eggs, such as mammalian cell culture, obviates egg adaptation and yields candidate vaccine viruses (CVVs) more antigenically similar to their original wild type viruses [5, 10]. Cell-based quadrivalent influenza vaccine (QIVc) grown in Madin-Darby canine kidney cell lines (Flucelvax Quadrivalent; CSL Seqirus USA Inc) first included a cell-derived CVV for the A(H3N2) strain during the 2017–2018 season. In the 2018–2019 season, QIVc included cell-derived CVVs for A(H3N2), B/Victoria, and B/Yamagata. In 2019–2020, all 4 CVVs within QIVc were cell derived, enabling isolation and vaccine manufacture entirely in Madin-Darby canine kidney lines [11].

Observational studies showed the benefit of cell- vs egg-based influenza vaccines during 3 seasons between 2017 and 2020 [12–18]. These studies used retrospective cohort designs with relative vaccine effectiveness (rVE) estimates based on influenza-related clinical diagnosis outcomes [12–18]. In addition, several test-negative design (TND) studies have reported the rVE of cell- vs egg-based influenza vaccines with test-confirmed influenza outcomes [19–23]. The TND studies evaluated vaccine effectiveness based on data from patients tested for influenza after presenting for medical care with respiratory illness symptoms [24, 25]. Because the controls met the same clinical criteria as cases, this design helped to reduce potential selection biases due to differential health care–seeking behavior. Furthermore, the use of test-confirmed influenza outcomes reduces the risk of misclassification of influenza status [24, 26]. The TND approach is often considered the best practice for prospective influenza surveillance systems and is increasingly being applied to retrospective studies [25, 27, 28].

Similar to the cohort studies cited previously, available TND studies have generally reported higher point estimates of cell- vs egg-based vaccine effectiveness [19–23]. However, none have had a sufficient sample size to reliably estimate rVE and with high precision. Therefore, using an integrated data set with a source population >120 million patients in the United States, we sought to retrospectively evaluate the rVE of QIVc vs egg-based quadrivalent influenza vaccine (QIVe) in preventing test-confirmed influenza in the outpatient setting over the 3 influenza seasons between 2017 and 2020.

## METHODS

We used an observational retrospective TND to estimate the rVE of QIVc vs QIVe in individuals aged 4 to 64 years against test-confirmed influenza during the 2017–2018, 2018–2019, and 2019–2020 influenza seasons in the United States. The study was designed, implemented, and reported in accordance with good pharmacoepidemiologic practice, applicable local regulations, and the ethical principles laid down in the Declaration of Helsinki as well as the RECORD recommendations (Reporting of Studies Conducted Using Observational Routinely Collected Health Data) [29, 30].

### Data Sources

Study data were sourced from the Veradigm Integrated Dataset [31]. Briefly, the data set links electronic health records (EHRs) held on the Veradigm Health Insights platform, which includes components from Allscripts Tiers 1 and 2 and Practice Fusion, with pharmacy and medical claims data from Komodo Health Inc. Veradigm is a large EHR company in the United States, with its data comprising primary care and other specialist interactions for >120 million patients. The Veradigm EHR platform serves primary care and other specialist physicians who provide a comprehensive array of health care services, including the issuing of prescriptions and vaccinations. Practices using the Veradigm EHR platform may elect to connect to a clinical laboratory electronically so that when a test result is available for a patient, it is sent directly into the EHR. Providers can also enter test results into the EHR manually. The data available on the results of the influenza tests performed as part of routine outpatient care form the basis of the outcome for this study. This study additionally used data from Komodo Health, a health care artificial intelligence company that collects, integrates, and licenses deidentified health care claims data. Administrative claims contain information about health care received in the inpatient and outpatient settings, as well as information about pharmacy prescriptions. Komodo sources data directly from payers as well as from broad-based health care sources, such as clearinghouses, pharmacies, and software platforms, and can capture a patient's activities regardless of the insurance provider. An “any claims” definition (open or closed) was used to conduct the analysis. Patient records from the multiple data sources are linked via patient-level deidentified tokens created deterministically from fields such as name, date of birth, and gender via an algorithm developed by Datavant. This integrated data set has been found to contain key variables for the assessment of influenza vaccine effectiveness and to be generally representative of the US insured population [31].

The database operates under certification of statistical deidentification according to US HIPAA regulations (Health Insurance Portability and Accountability Act) by a third-party HIPAA expert determination provider. The certification process ensures that investigators have access only to deidentified data and cannot pose a risk to reidentification; studies based on such certification are therefore exempt from institutional review board approval and from obtaining informed consent.

The study included 3 influenza seasons in the United States (Centers for Disease Control and Prevention [CDC] epidemiology weeks 40 through 20): 1 October 2017 to 19 May 2018, 30 September 2018 to 18 May 2019, and 29 September 2019 to 7 March 2020. The last season was truncated to March 2020 because health care–seeking behavior and influenza circulation were dramatically altered with the onset of the COVID-19 pandemic [32].

### Patient Consent Statement

This study does not include factors necessitating patient consent.

### Study Population

The study population comprised vaccinated patients aged 4 to 64 years who were tested for influenza as part of routine care within 7 days of presenting with an acute respiratory or febrile illness (ARFI). ARFI was identified by *ICD-10-CM* diagnosis codes (Supplementary Table 1) [33]. In each season, eligible participants were vaccinated with either QIVc or QIVe between 1 August and 14 days before they were tested for influenza. They also had EHR transcript and claims activity spanning ≥1 year prior to the vaccination date so that baseline demographic and clinical data could be captured. Individuals were excluded if data for sex and geographic region were missing in the EHR, as these were considered a proxy for data quality and completeness.

Vaccination status was identified by CVX codes (vaccine administered), *CPT* codes, and National Drug Codes (Supplementary Table 2). Participants were excluded if (1) QIVc or QIVe was received <14 days prior to testing; (2) they received any influenza vaccination between the end of the previous influenza season (as defined by the CDC) and 1 August of the current season; and (3) they had >1 influenza immunization recorded between 1 August and the influenza test date during the same season or, if aged <9 years, had >2 influenza vaccines or 2 different influenza vaccines (ie, a mix of QIVc and QIVe) between 1 August and the influenza test date.

Influenza testing information was identified via LOINC (Logical Observation Identifiers Names and Codes), *CPT*, or Systematized Nomenclature of Medicine concepts (Supplementary Table 3). All types of influenza test were considered: antigen, molecular (polymerase chain reaction, nucleic acid test), virus culture, and antibody. The first influenza test result was considered for each season, except when a negative test result preceded a positive one during the season; the positive test result was considered in keeping with the principle of cumulative seasonal incidence. Individuals testing positive were classified as “cases” while those testing negative were classified as “controls” (Supplementary Text 1). The date of the valid influenza test result (definitively positive or negative) was defined as the test date (Supplementary Text 2).

### Statistical Analyses

For a complete description of the statistical methods, see Supplementary Text 3 and Supplementary Tables 4 and 5.

A logistic regression model was used to obtain odds ratios comparing the odds of testing positive for influenza between QIVc and QIVe recipients for each season. Influenza status was modeled as the outcome and vaccination type (QIVc vs QIVe) as the exposure. rVE was calculated by the formula rVE = (1 – odds ratio) × 100% and reported with 95% CIs. The main analysis is based on a doubly robust approach combining inverse probability of treatment weighting (IPTW) and multivariable adjustment. Results of the stepwise implementation of the model are reported in the supplementary materials. These include an unadjusted rVE, a model adjusted with IPTW only, and a standard multivariable model only (Supplementary Table 4).

The methodology used to perform the doubly robust analysis is detailed in Supplementary Text 3. Briefly, the propensity score models used to derive the IPTW weights were adjusted for the covariates defined a priori—age (continuous), sex, region (Northeast, Midwest, South, West), and influenza test date (as spline)—and any other covariates shown to be unbalanced between vaccine exposure groups before weighting. Other assessed covariates included race, ethnicity, week of vaccination, Charlson Comorbidity Index (CCI) score [34], the presence of high-risk medical conditions for which the CDC recommends influenza vaccination, and health care resource utilization (Supplementary Table 6). In a TND study, the control group is assumed to represent the overall source-eligible population. It is therefore within this control group that characteristics of patients in each vaccine group are evaluated for potential confounding. Covariate balance between vaccine exposure groups was evaluated among the controls by standardized mean differences (SMDs), with an a priori threshold of an absolute value ≤0.1 indicating a negligible difference. Using established methods for estimating propensity scores within case-control studies [35], we first estimated propensity scores for treatment group membership (ie, QIVc vs QIVe) among controls and then used the fitted model to calculate propensity for treatment group membership scores for the cases. Propensity scores were used to calculate stabilized weights. For the doubly robust analysis, the IPTW sample was used in a multivariable logistic regression model adjusted for the a priori covariates age, sex, region and test date and any other covariates that remained unbalanced after weighting (Supplementary Table 5).

Three prespecified sensitivity analyses were conducted to evaluate the robustness of key assumptions and residual confounding in the main analysis. First, the “propensity to be tested” was assessed to address the potential bias inherent in the selection of patients who were tested for influenza, regardless of vaccine type. Second, the analysis was limited to the seasonal peak period to examine potential bias from the lower predictive performance of influenza tests when influenza activity is low. The peak period was determined by the moving epidemic method algorithm for each season [36]. Epidemic thresholds for the start and stop of the influenza circulation were calculated per CDC laboratory data on the percentage of outpatient influenza tests administered that were positive for influenza [37, 38]. This restricted the analyses to 11 December 2017 to 18 March 2018, 17 December 2018 to 7 April 2019, and 8 December 2019 to 7 March 2019. Third, “matched index week”—which matched cases and controls who were tested for influenza during the same week of the calendar year—was used to address potential residual confounding due to the timing of the test, since this approach is less sensitive to model misspecification than the adjustment in the main analysis [39, 40]. Further sensitivity analysis excluding antibody and culture tests was performed as recommended during the peer review process, to address potential bias due to the lack of specificity for antibody tests to reliably detect acute disease and potential differences in patients tested by culture methods.

An exploratory sensitivity analysis examined the potential impact of the sensitivity and specificity of the influenza tests, and 2 post hoc analyses were conducted to explore observed differences in rVE estimates derived from the multivariable regression relative to the doubly robust analysis in the 2019–2020 season. Methods are described in Supplementary Text 3.

## RESULTS

### Study Population

**Figure 1.**
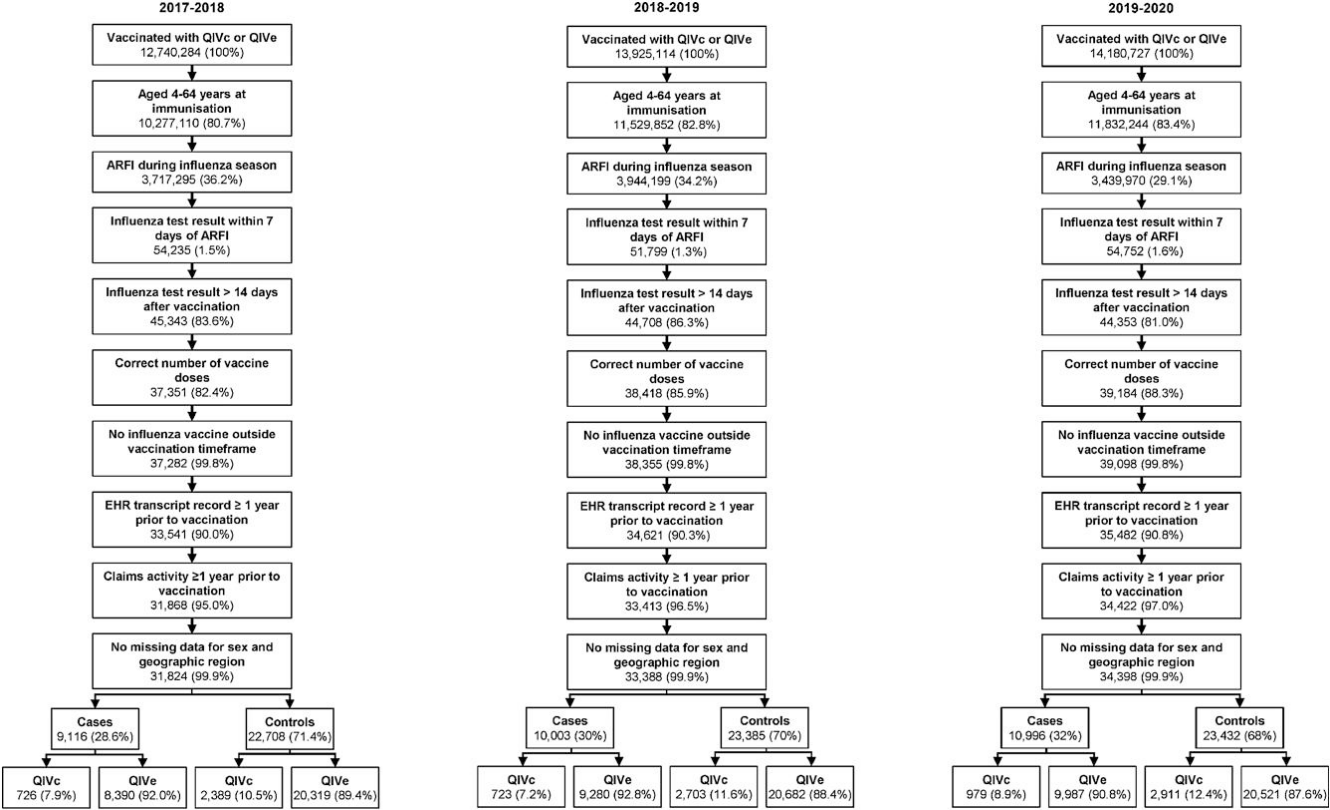
Study population selection for each season. ARFI, acute respiratory or febrile illness; EHR, electronic health record; QIVc, cell-based quadrivalent influenza vaccine; QIVe, egg-based quadrivalent influenza vaccine.

**Figure 2.**
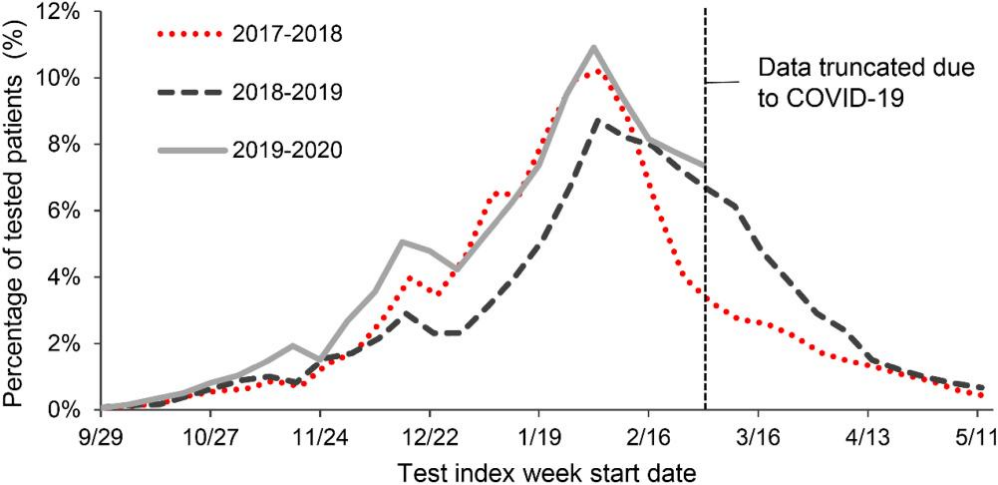
Percentage of study populations tested for influenza within 7 days of a documented acute respiratory or febrile illness. From the influenza seasons of 2017–2018 (dotted line), 2018–2019 (dashed line), and 2019–2020 (solid line). Data from the final season were truncated to avoid confounding factors due to onset of the COVID-19 pandemic.

The selection of the study population based on inclusion and exclusion criteria is presented for each season in Figure 1 and Supplementary Table 7. The final study populations included 31 824 individuals in the 2017–2018 season, 33 388 in 2018–2019, and 34 398 in 2019–2020. Of these, 3115 (9.8%), 3426 (10.3%), and 3890 (11.3%) were respectively vaccinated with QIVc and 28 709 (90.2%), 29 962 (89.7%), and 30 508 (88.7%) with QIVe (Supplementary Table 7). Over all 3 seasons, influenza testing increased gradually in October and November, accelerated in December and January, and peaked in late January and early February (Figure 2). For 2019–2020, the full curve could not be observed due to the COVID-19 pandemic, but the initial trends were similar to the 2017–2018 season.

Across the 3 seasons, in the unweighted population, the mean ages of QIVc recipients were 42.5, 38.8, and 37.2 years, whereas the mean ages of QIVe recipients were 27.6, 23.3, and 21.8 years (Supplementary Table 6). The majority of QIVe recipients in each season were aged 4 to 17 years, while most QIVc recipients were adults. In each season, the majority of participants were female, White, non-Hispanic, and from southern US states. The mean ± SD CCI score ranged from 0.5 ± 1.0 in 2019–2020 to 0.6 ± 1.2 in 2017–2018 among QIVc recipients and from 0.3 ± 0.8 in 2019–2020 to 0.5 ± 1.0 in 2017–2018 in the QIVe group. The QIVc group included more individuals with ≥1 high-risk medical condition (Supplementary Figure 1; Supplementary Table 6). Baseline all-cause health care resource utilization was similar between vaccine groups, with an average of 5.3 to 5.7 outpatient visits in the 12 months prior to vaccination in each season. Across all seasons, 5.0% to 6.5% of patients had at least 1 inpatient admission, while 17.7% to 19.6% had at least 1 emergency department visit (Supplementary Table 6).

**Figure 3.**
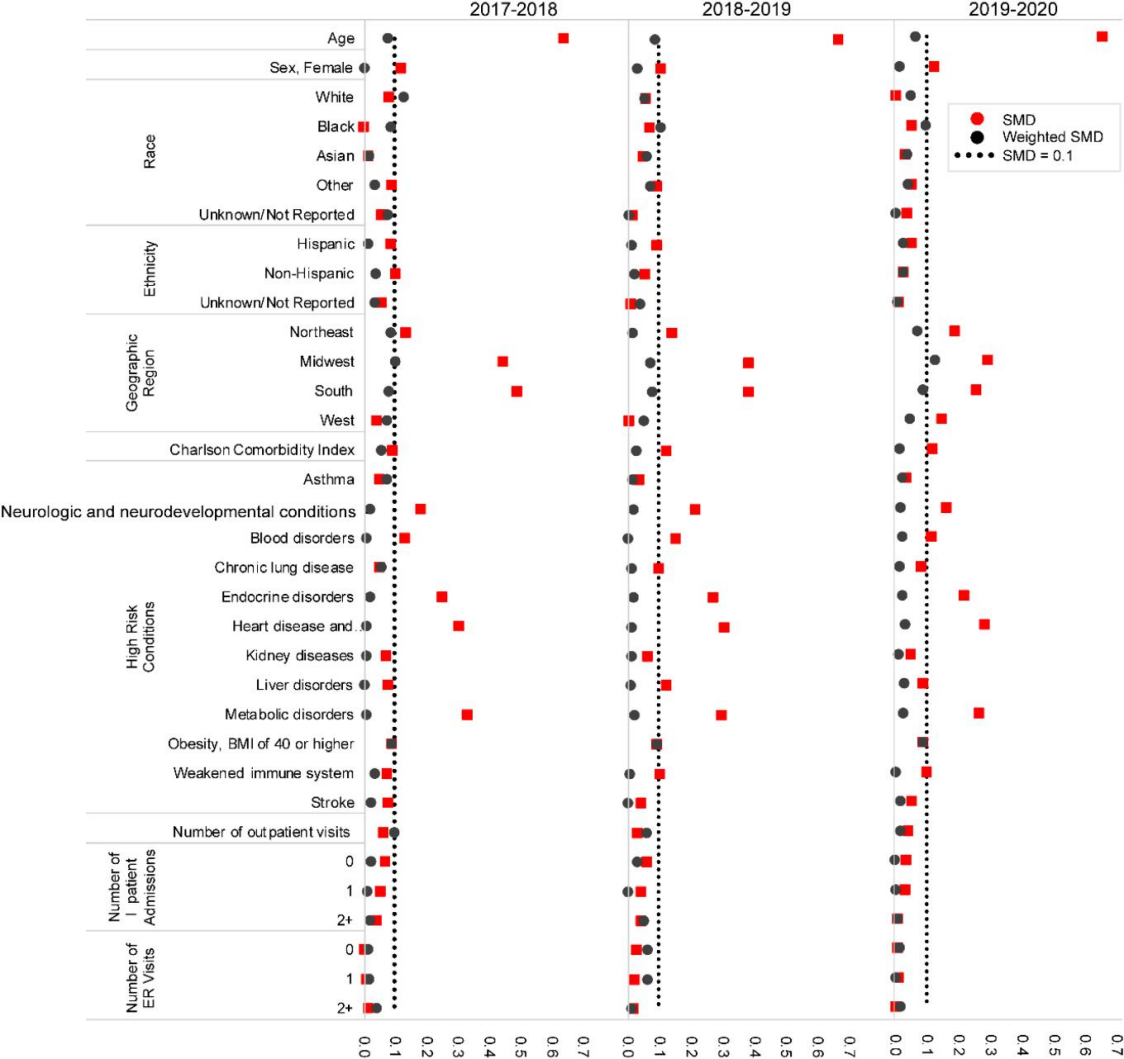
Covariate balance of control population before (squares) and after (dots) inverse probability of treatment weighting. Standardized mean difference (SMD) values ≤0.1 (dotted line) indicate a negligible difference.

Prior to weighting, there was an imbalance (SMD >|0.1|) between the QIVc controls and QIVe controls in all seasons across age, region, and 5 of 12 high-risk conditions (neurologic and neurodevelopmental conditions, blood disorders, endocrine disorders, heart disease and related conditions, and metabolic disorders), as well as season-specific imbalances in sex in the 2017–2018 and 2019–2020 seasons, West region in the 2019–2020 season, CCI in the 2018–2019 and 2019–2020 seasons, and liver disorders in the 2018–2019 season. After IPTW, covariate balance was achieved in all 3 seasons, with SMDs ≤|0.1| for each covariate except White race (SMD, 0.13) during the 2017–2018 season and Midwest region (SMD, 0.13) during the 2019–2020 season (Figure 3).

Most influenza testing was done with an antigen test (>90% of testing in the first and second seasons and >80% in the third) or with a molecular test (6%–18% across seasons). Culture and antibody tests each accounted for <1% of samples (Supplementary Tables 6 and 8).

Among QIVc recipients, 23.3%, 21.1%, and 25.2% of each season's study population had a test-confirmed case of influenza, as compared with 29.2%, 31.0%, and 32.7% of QIVe recipients (Supplementary Table 7).

### rVE of QIVc vs QIVe

**Figure 4.**
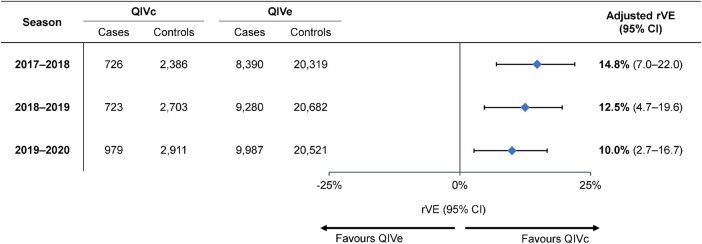
Adjusted relative vaccine effectiveness (rVE) of cell-based quadrivalent influenza vaccine (QIVc) vs egg-based quadrivalent influenza vaccine (QIVe) over 3 influenza seasons in a doubly robust analysis based on inverse probability of treatment weighting.

In the doubly robust analysis, the estimated rVE of QIVc vs QIVe was 14.8% (95% CI, 7.0%–22.0%), 12.5% (95% CI, 4.7%–19.6%), and 10.0% (95% CI, 2.7%–16.7%) in the 2017–2018, 2018–2019, and 2019–2020 seasons, respectively (Figure 4; Supplementary Table 9).

**Figure 5.**
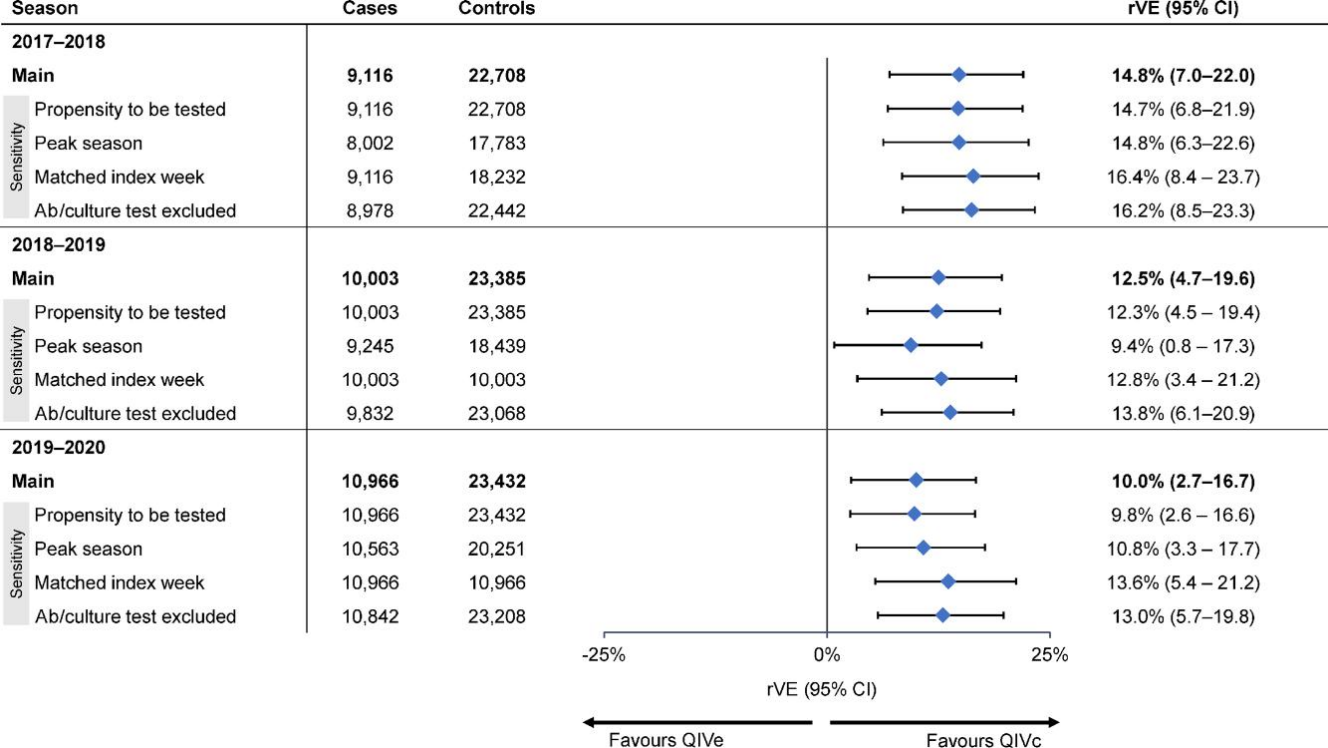
Sensitivity analyses of relative vaccine effectiveness (rVE) of cell-based quadrivalent influenza vaccine (QIVc) vs egg-based quadrivalent influenza vaccine (QIVe) by (1) propensity to be tested for influenza, (2) seasonal peak period, (3) matched index week (ie, individuals tested for influenza during the same week of the calendar year), and (4) exclusion of antibody (Ab) and culture tests for influenza.

In each season, the rVE point estimates and 95% CIs of sensitivity analyses based on (1) propensity to be tested for influenza, (2) peak influenza season, (3) matched index week (patients tested for influenza during the same week of the calendar year), and (4) exclusion of culture and antibody tests were within 2% to 3% of the main doubly robust analysis results, with overlapping 95% CIs that excluded the null (Figure 5, Supplementary Table 10).

Results of the unadjusted model, the model adjusted with IPTW only, and the standard multivariable model only are shown in Supplementary Table 9. Results of the exploratory and post hoc analyses are shown in Supplementary Tables 11 and 12.

## DISCUSSION

Over 3 seasons evaluated by a retrospective TND approach, QIVc was superior to QIVe in preventing test-confirmed influenza among individuals aged 4 to 64 years. Our estimated rVEs of QIVc vs QIVe were 14.8% (7.0%–22.0%), 12.5% (4.7%–19.6%), and 10.0% (2.7%–16.7%) during the 2017–2018, 2018–2019, and 2019–2020 influenza seasons, respectively. Sensitivity analyses examining the impact of propensity to be tested, restriction to the peak influenza season, and matching on test week resulted in rVE point estimates and 95% CIs that were consistent with the main findings, supporting their robustness.

The improved effectiveness of QIVc over QIVe observed in this study is consistent with the expected advantages of propagation of human influenza viruses in qualified mammalian cell lines, obviating the need for egg-adapted mutations and doubling the rate of virus isolation as compared with egg-based propagation, thus improving the choice, match, and potentially the effectiveness of seasonal influenza vaccines as compared with egg-based vaccines [5]. For the first time, in 2017–2018 the World Health Organization provided a recommendation for a cell-derived CVV for the A(H3N2) strain that was included in QIVc, while the other strains remained egg derived (Supplementary Table 13). Egg adaptation in the A(H3N2) vaccine virus was considered a major cause of antigenic mismatch and reduced vaccine effectiveness in this season, with the egg-based CVV (A/Hong Kong/4801/2014) having acquired the T160K, L94P, and N96S egg-adaptive mutations [5]. The T160K and L194P mutations are located in antigenic site B of the H3 HA protein and have been shown to alter the antigenicity of the vaccine through loss of a glycosylation site and altered mobility [7, 9]. The US 2018–2019 season initially had predominant circulation of A(H1N1) virus, followed by cocirculation with A(H3N2) virus, which drifted away from the vaccine strain toward the end of the season [41]. In this season, the A(H3N2) and both influenza B CVVs for the QIVc were cell derived (Supplementary Table 13). Egg adaptation and drift contributed to the low observed vaccine effectiveness, with the recommended egg-based CVV (A/Singapore/INFIMH-16-0019/2016) having acquired the same T160K and L94P egg-adaptive mutations as the previous season's vaccine virus, as well as D225G, a substitution located in the receptor-binding site [5, 6]. The 2019–2020 season was characterized by predominant circulation of B/Victoria and A(H1N1) viruses [42]. At that point, all CVVs for QIVc were cell derived. Antigenic characterization showed fewer egg-propagated B/Victoria viruses that were antigenically similar to the reference virus as compared with cell-propagated viruses (8% vs 60%), suggesting egg adaptation [43]. Indeed, N195T and T197I substitutions (vs the original clinical specimen) were observed in the HA proteins of the egg-based CVVs B/Colorado/06/2017 and B/Maryland/15/2016, respectively (K. Laurie, personal communication). Both these substitutions are found in the 190-helix antigenic site of the influenza B HA [5, 44]. The A(H1N1) egg-based CVV A/Brisbane/02/2018 contained a Q223R adaptation, and the egg-based CVV A/Switzerland/3330/2017 contained K209M and E224K adaptations in the HA protein as compared with cell-grown viruses (K. Laurie, personal communication). Q223R and E224K are located in the receptor-binding site of the H1 HA [5, 45]. The A(H1N1) virus in the vaccine was antigenically similar to the predominant circulating A(H1N1) virus according to antigenic characterization with ferret antisera [46]. However, assays based on postvaccination human antisera showed that circulating A(H1N1) viruses had a decreased antigenic similarity to the cell-propagated reference virus and even more pronounced differences when compared with egg-propagated A(H1N1) viruses, suggesting the potential impact of egg adaptation in combination with drift [47]. Taken together, our findings of a benefit of QIVc in all seasons align with the characteristics and predominance of the circulating strains during these seasons.

The findings of the present retrospective TND study are consistent with prior observational studies evaluating the rVE of QIVc vs QIVe over the 3 seasons between 2017 and 2020 [12–23, 48, 49]. The point estimates in these studies favored QIVc over QIVe, except for a retrospective TND conducted by Tseng et al during the 2018–2019 season among <1500 participants [23]. With an average of 33 000 participants in 3 consecutive seasons, the current study is the largest available one to use test-confirmed outcomes for this comparison between QIVc and QIVe. Findings from the current study corroborate the results of large retrospective cohort studies (population size ≥1 million) that were based on clinical diagnosis [12–17, 48]. Also similar to the previous findings was the observation of a more pronounced benefit during the 2017–2018 season when egg adaptation occurred for the predominant circulating strain, with no evidence of drift.

This study had several strengths. In prospective TND studies, all eligible patients meet a clinical case definition and are tested for influenza. In our study, we followed this principle by limiting the sample to patients who had an ARFI diagnosis within 7 days of an influenza test—an indication that the test was performed because of the influenza-like symptoms. This approach produces a more clinically homogeneous study population, which helps to eliminate potential bias due to differences between vaccination groups in terms of severity of disease that would prompt patients to seek medical care and testing [50]. Another strength, which is common to all TND studies, is the focus on test-confirmed outcomes rather than clinical diagnoses of influenza that may or may not have been informed by an influenza test. The large data set included patients from across the United States as well as data from 3 influenza seasons. The latter is critical given the variation of seasonal characteristics. Furthermore, the completeness of the data set allowed for the adjustment of several well-established confounders, such as age, sex, calendar time, region, and other clinical and demographic characteristics. Exposure, outcome, and covariate information was ascertained retrospectively from patient records in the same manner for all exposure populations.

When compared with traditional observational studies, the TND may reduce, but does not remove, confounding and selection bias due to differential health care–seeking behaviors. Furthermore, because utilization of US health care resources is intermittent or opportunistic, the amount and quality of data available on individuals may vary and result in bias if dependent on type of vaccination received. The type of vaccine received could also be a proxy for health system/health care access differences that could lead to differential utilization. However, the requirement for claims activity spanning ≥1 year prior to the vaccination date ensured that the full study population was insured and had access to health care prior to its ARFI diagnosis, and baseline health care–seeking behavior was comparable between vaccine groups, minimizing potential residual confounding due to differences in health care access and health care–seeking behavior. In this retrospective TND study, influenza test confirmation was obtained as part of routine care and not performed according to preset screening criteria, although the latter was approximated by the requirement that patients have a diagnosis of ARFI in temporal proximity to influenza testing. A “propensity to be tested” sensitivity analysis addressed the potential bias of which patients are given an influenza test. Another limitation is that the accuracy of the test-negative approach is affected by the sensitivity and specificity of the diagnostic tests used to determine case status. However, studies have shown that imperfect specificity causes greater bias than imperfect sensitivity, and confidence is provided by the Food and Drug Administration requirement for rapid antigen tests to demonstrate at minimum 95% sensitivity and 80% sensitivity for detection of influenza A and B as compared with reverse transcription polymerase chain reaction tests [51, 52]. This was confirmed in our study by findings from an exploratory analysis, which found that correction for potential misclassification bias introduced by test sensitivity and specificity appeared to have minimal impact on the rVE estimates. Finally, because vaccination was not randomly assigned, residual and unmeasured confounding remains as a potential source of bias. The methodology used in this study leveraged available data, but clinical and claims data sources can be incomplete (eg, race and ethnicity) and do not include individual or contextual socioeconomic data that could inform health-seeking behavior and affect generalizability to the noninsured population. Additionally, the data assets lacked information on influenza tests administered in a hospital.

In conclusion, we found that QIVc was more effective than QIVe at preventing test-confirmed influenza infections during the 2017–2018, 2018–2019, and 2019–2020 seasons, which were characterized by different circulating viruses and degrees of egg adaptation.

## Supplementary Material

ofae175_Supplementary_Data

## Supplementary Data

Supplementary materials are available at *Open Forum Infectious Diseases* online. Consisting of data provided by the authors to benefit the reader, the posted materials are not copyedited and are the sole responsibility of the authors, so questions or comments should be addressed to the corresponding author.

## Notes

***Acknowledgments***. We thank Dr Karen Laurie, CSL Seqirus, Melbourne, Australia, for her advice and input on the egg-adaptive mutations found in the egg-based CVVs. Medical consultant C. Gordon Beck and Amanda M. Justice provided editorial and medical writing assistance, which was funded by CSL Seqirus.

***Author contributions***. All authors were involved in study conception and design. C. W. M., A. D., and A. N. B. conducted the analysis. All authors were involved in the interpretation of data. A. N. S., C. W. M., and K. W. M. were involved in drafting the manuscript, and I. M., A. D., A. N. B., S. G. S., and M. D. M. H. revised it critically. All authors made substantive intellectual contributions to the development of this manuscript and approved the final version.

***Data availability*.** The data sets used in this study are privately licensed and are not available to maintain patient privacy.

***Financial support***. This work was supported by CSL Seqirus.
