# Supplementary material for: Relative Vaccine Effectiveness of Cell- vs Egg-Based Quadrivalent Influenza Vaccine Against Test-Confirmed Influenza Over 3 Seasons Between 2017 and 2020 in the United States
Source: Open Forum Infect Dis. 2024 May 2;11(5):ofae175. doi: 10.1093/ofid/ofae175 (PMC11064727; doi:10.1093/ofid/ofae175)
Supplement: ofae175_Supplementary_Data [file ofae175_supplementary_data.docx]

**SUPPLEMENTARY MATERIALS FOR:**

**Relative vaccine effectiveness of cell-based versus egg-based quadrivalent influenza vaccine against test-confirmed influenza over 3 seasons between 2017 and 2020**

**Table of Contents**

[Supplementary Table 1. Acute respiratory or febrile illness codes 3](#_Toc160456533)

[Supplementary Table 2. CVX, CPT, and NDC codes for influenza vaccines 8](#_Toc160456534)

[Supplementary Table 3. Influenza test codes 9](#_Toc160456535)

[Supplementary Text 1. Case definitions 15](#_Toc160456536)

[Supplementary Text 2. Mapping of structured and unstructured data to identify test results 16](#_Toc160456537)

[Supplementary Text 3. Statistical methodology 17](#_Toc160456538)

[Supplementary Table 4. Summary of statistical analyses 24](#_Toc160456539)

[Supplementary Table 5. Final covariates in adjusted statistical models of the primary analyses 26](#_Toc160456540)

[Supplementary Table 6. Demographic and clinical characteristics of the study population at index date, irrespective of case status 27](#_Toc160456541)

[Supplementary Table 7. Study population selection in each season 29](#_Toc160456542)

[Supplementary Figure 1. Proportions of test-positive influenza case and control study populations with at least 1 high-risk medical condition within each vaccine group during each season 30](#_Toc160456543)

[Supplementary Table 8. Demographic characteristics of the study population at index date by case and control populations within each vaccine group 31](#_Toc160456544)

[Supplementary Table 9. Primary analyses: rVEs of QIVc versus QIVe against positive influenza test result of the full study population, by season 35](#_Toc160456545)

[Supplementary Table 10. Sensitivity analyses: rVEs of QIVc versus QIVe against positive influenza test result of the full study population, by season 36](#_Toc160456546)

[Supplementary Table 11. Exploratory multivariable regression sensitivity analysis: rVEs of QIVc versus QIVe against positive influenza test result of the antigen and molecular test population, by season 37](#_Toc160456547)

[Supplementary Table 12. Post hoc multivariable regression analysis: rVEs of QIVc versus QIVe against positive influenza test result of the matched on week population, by season 38](#_Toc160456548)

[Supplementary Table 13. Influenza virus strains included in QIVc and QIVe over the study period 39](#_Toc160456549)

[References 40](#_Toc160456550)

Supplementary Table 1. Acute respiratory or febrile illness codes

Note: The gray shading in the table is used to separate groups of codes (e.g., J01.x codes vs J02.x codes).

| **Acute respiratory or febrile illness** [1] | **ICD-10-CM code** [1] |
| --- | --- |
| Sepsis, unspecified organism | A41.9 |
| Viral infection of unspecified site | B34 |
| Adenovirus infection, unspecified | B34.0 |
| Enterovirus infection, unspecified | B34.1 |
| Coronavirus infection, unspecified | B34.2 |
| Parvovirus infection, unspecified | B34.3 |
| Papovavirus infection, unspecified | B34.4 |
| Other viral infection, unspecified | B34.8 |
| Viral infection, unspecified | B34.9 |
| Respiratory syncytial virus as the cause of diseases classified elsewhere | B97.4 |
| Other viral agents as the cause of diseases classified elsewhere | B97.8 |
| Human metapneumovirus as the cause of diseases classified elsewhere | B97.81 |
| Other viral agents as the cause of diseases classified elsewhere | B97.89 |
| Acute nasopharyngitis | J00 |
| Acute sinusitis | J01 |
| Acute maxillary sinusitis | J01.0 |
| Acute maxillary sinusitis, unspecified | J01.00 |
| Acute recurrent maxillary sinusitis | J01.01 |
| Acute frontal sinusitis | J01.1 |
| Acute frontal sinusitis, unspecified | J01.10 |
| Acute recurrent frontal sinusitis | J01.11 |
| Acute ethmoidal sinusitis | J01.2 |
| Acute ethmoidal sinusitis, unspecified | J01.20 |
| Acute recurrent ethmoidal sinusitis | J01.21 |
| Acute sphenoidal sinusitis | J01.3 |
| Acute sphenoidal sinusitis, unspecified | J01.30 |
| Acute recurrent sphenoidal sinusitis | J01.31 |
| Acute pansinusitis | J01.4 |
| Acute pansinusitis, unspecified | J01.40 |
| Acute recurrent pansinusitis | J01.41 |
| Other acute sinusitis | J01.8 |
| Other acute sinusitis | J01.80 |
| Other acute recurrent sinusitis | J01.81 |
| Other acute sinusitis | J01.9 |
| Acute sinusitis, unspecified | J01.90 |
| Acute recurrent sinusitis, unspecified | J01.91 |
| Acute pharyngitis | J02 |
| Streptococcal pharyngitis | J02.0 |
| Acute pharyngitis due to other specified organisms | J02.8 |
| Acute pharyngitis, unspecified | J02.9 |
| Acute tonsillitis | J03 |
| Streptococcal tonsillitis | J03.0 |
| Acute streptococcal tonsillitis, unspecified | J03.00 |
| Acute recurrent streptococcal tonsillitis | J03.01 |
| Acute tonsillitis due to other specified organisms | J03.8 |
| Acute tonsillitis due to other specified organisms | J03.80 |
| Acute recurrent tonsillitis due to other specified organisms | J03.81 |
| Acute tonsillitis, unspecified | J03.9 |
| Acute tonsillitis, unspecified | J03.90 |
| Acute recurrent tonsillitis, unspecified | J03.91 |
| Acute laryngitis and tracheitis | J04 |
| Acute laryngitis | J04.0 |
| Acute tracheitis | J04.1 |
| Acute tracheitis without obstruction | J04.10 |
| Acute tracheitis with obstruction | J04.11 |
| Acute laryngotracheitis | J04.2 |
| Supraglottitis, unspecified | J04.3 |
| Supraglottitis, unspecified, without obstruction | J04.30 |
| Supraglottitis, unspecified, with obstruction | J04.31 |
| Acute upper respiratory infections of multiple and unspecified sites | J06 |
| Acute laryngopharyngitis | J06.0 |
| Other acute upper respiratory infections of multiple sites | J06.8 |
| Acute upper respiratory infection, unspecified | J06.9 |
| Influenza due to certain identified influenza viruses | J09 |
| Influenza due to identified novel influenza A virus | J09.X |
| Influenza due to identified novel influenza A virus with pneumonia | J09.X1 |
| Influenza due to identified novel influenza A virus with other respiratory manifestations | J09.X2 |
| Influenza due to identified novel influenza A virus with gastrointestinal manifestations | J09.X3 |
| Influenza due to identified novel influenza A virus with other manifestations | J09.X9 |
| Influenza due to other identified influenza virus | J10 |
| Influenza due to other identified influenza virus with pneumonia | J10.0 |
| Influenza due to other identified influenza virus with unspecified type of pneumonia | J10.00 |
| Influenza due to other identified influenza virus with the same other identified influenza virus pneumonia | J10.01 |
| Influenza due to other identified influenza virus with other specified pneumonia | J10.08 |
| Influenza due to other identified influenza virus with other respiratory manifestations | J10.1 |
| Influenza due to other identified influenza virus with gastrointestinal manifestations | J10.2 |
| Influenza due to other identified influenza virus with other manifestations | J10.8 |
| Influenza due to other identified influenza virus with encephalopathy | J10.81 |
| Influenza due to other identified influenza virus with myocarditis | J10.82 |
| Influenza due to other identified influenza virus with otitis media | J10.83 |
| Influenza due to other identified influenza virus with other manifestations | J10.89 |
| Influenza due to unidentified influenza virus | J11 |
| Influenza due to unidentified influenza virus with pneumonia | J11.0 |
| Influenza due to unidentified influenza virus with unspecified type of pneumonia | J11.00 |
| Influenza due to unidentified influenza virus with specified pneumonia | J11.08 |
| Influenza due to unidentified influenza virus with other respiratory manifestations | J11.1 |
| Influenza due to unidentified influenza virus with gastrointestinal manifestations | J11.2 |
| Influenza due to unidentified influenza virus with other manifestations | J11.8 |
| Influenza due to unidentified influenza virus with encephalopathy | J11.81 |
| Influenza due to unidentified influenza virus with myocarditis | J11.82 |
| Influenza due to unidentified influenza with otitis media | J11.83 |
| Influenza due to unidentified influenza virus with other manifestations | J11.89 |
| Viral pneumonia, not elsewhere classified | J12 |
| Adenoviral pneumonia | J12.0 |
| Respiratory syncytial virus pneumonia | J12.1 |
| Parainfluenza virus pneumonia | J12.2 |
| Human metapneumovirus pneumonia | J12.3 |
| Other viral pneumonia | J12.8 |
| Pneumonia due to SARS-associated coronavirus | J12.81 |
| Other viral pneumonia | J12.89 |
| Viral pneumonia, unspecified | J12.9 |
| Pneumonia due to Streptococcus pneumoniae | J13 |
| Pneumonia due to Hemophilus influenzae | J14 |
| Bacterial pneumonia, not elsewhere classified | J15 |
| Pneumonia due to Klebsiella pneumoniae | J15.0 |
| Pneumonia due to Pseudomonas | J15.1 |
| Pneumonia due to staphylococcus | J15.2 |
| Pneumonia due to staphylococcus, unspecified | J15.20 |
| Pneumonia due to staphylococcus aureus | J15.21 |
| Pneumonia due to Methicillin susceptible Staphylococcus aureus | J15.211 |
| Pneumonia due to Methicillin resistant Staphylococcus aureus | J15.212 |
| Pneumonia due to other staphylococcus | J15.29 |
| Pneumonia due to streptococcus, group B | J15.3 |
| Pneumonia due to other streptococci | J15.4 |
| Pneumonia due to Escherichia coli | J15.5 |
| Pneumonia due to other aerobic Gram-negative bacteria | J15.6 |
| Pneumonia due to Mycoplasma pneumoniae | J15.7 |
| Pneumonia due to other specified bacteria | J15.8 |
| Unspecified bacterial pneumonia | J15.9 |
| Pneumonia due to other infectious organisms, not elsewhere classified | J16 |
| Chlamydial pneumonia | J16.0 |
| Pneumonia due to other specified infectious organisms | J16.8 |
| Pneumonia in diseases classified elsewhere | J17 |
| Pneumonia, unspecified organism | J18 |
| Bronchopneumonia, unspecified organism | J18.0 |
| Lobar pneumonia, unspecified organism | J18.1 |
| Hypostatic pneumonia, unspecified organism | J18.2 |
| Other pneumonia, unspecified organism | J18.8 |
| Pneumonia, unspecified organism | J18.9 |
| Acute bronchitis | J20 |
| Acute bronchitis due to Mycoplasma pneumoniae | J20.0 |
| Acute bronchitis due to Hemophilus influenzae | J20.1 |
| Acute bronchitis due to streptococcus | J20.2 |
| Acute bronchitis due to coxsackievirus | J20.3 |
| Acute bronchitis due to parainfluenza virus | J20.4 |
| Acute bronchitis due to respiratory syncytial virus | J20.5 |
| Acute bronchitis due to rhinovirus | J20.6 |
| Acute bronchitis due to echovirus | J20.7 |
| Acute bronchitis due to other specified organisms | J20.8 |
| Acute bronchitis, unspecified | J20.9 |
| Acute bronchiolitis | J21 |
| Acute bronchiolitis due to respiratory syncytial virus | J21.0 |
| Acute bronchiolitis due to human metapneumovirus | J21.1 |
| Acute bronchiolitis due to other specified organisms | J21.8 |
| Acute bronchiolitis, unspecified | J21.9 |
| Unspecified acute lower respiratory infection | J22 |
| Other specified diseases of upper respiratory tract | J39.8 |
| Disease of upper respiratory tract, specified | J39.9 |
| Bronchitis, not specified as acute or chronic | J40 |
| Acute respiratory distress syndrome | J80 |
| Pulmonary edema | J81 |
| Acute pulmonary edema | J81.0 |
| Chronic pulmonary edema | J81.1 |
| Pleural effusion, not elsewhere classified | J90 |
| Acute respiratory failure | J96.0 |
| Acute respiratory failure, unspecified whether with hypoxia or hypercapnia | J96.00 |
| Acute respiratory failure with hypoxia | J96.01 |
| Acute respiratory failure with hypercapnia | J96.02 |
| Acute and chronic respiratory failure | J96.2 |
| Acute and chronic respiratory failure, unspecified whether with hypoxia or hypercapnia | J96.20 |
| Acute and chronic respiratory failure with hypoxia | J96.21 |
| Acute and chronic respiratory failure with hypercapnia | J96.22 |
| Respiratory failure, unspecified, unspecified | J96.9 |
| Respiratory failure, unspecified, unspecified whether with hypoxia or hypercapnia | J96.90 |
| Respiratory failure, unspecified with hypoxia | J96.91 |
| Respiratory failure, unspecified with hypercapnia | J96.92 |
| Other diseases of bronchus, not elsewhere classified | J98.09 |
| Pulmonary collapse | J98.1 |
| Atelectasis | J98.11 |
| Other pulmonary collapse | J98.19 |
| Other disorders of lung | J98.4 |
| Other specified respiratory disorders | J98.8 |
| Respiratory disorder, unspecified | J98.9 |
| Shock during or following labor and delivery | O75.1 |
| Pyrexia during labor, not elsewhere classified | O75.2 |
| Pyrexia of unknown origin following delivery | O86.4 |
| Other viral diseases complicating pregnancy/childbirth | O98.5 |
| Other viral diseases complicating pregnancy | O98.51 |
| Other viral diseases complicating pregnancy, first trimester | O98.511 |
| Other viral diseases complicating pregnancy, second trimester | O98.512 |
| Other viral diseases complicating pregnancy, third trimester | O98.513 |
| Other viral diseases complicating pregnancy, unspecified trimester | O98.519 |
| Other viral diseases complicating childbirth | O98.52 |
| Other viral diseases complicating the puerperium | O98.53 |
| Other maternal infectious and parasitic diseases complicating pregnancy | O98.81 |
| Other maternal infectious and parasitic diseases complicating pregnancy, first trimester | O98.811 |
| Other maternal infectious and parasitic diseases complicating pregnancy, second trimester | O98.812 |
| Other maternal infectious and parasitic diseases complicating pregnancy, third trimester | O98.813 |
| Other maternal infectious and parasitic diseases complicating pregnancy, unspecified trimester | O98.819 |
| Diseases of the respiratory system complicating pregnancy | O99.51 |
| Diseases of the respiratory system complicating pregnancy, first trimester | O99.511 |
| Diseases of the respiratory system complicating pregnancy, second trimester | O99.512 |
| Diseases of the respiratory system complicating pregnancy, third trimester | O99.513 |
| Diseases of the respiratory system complicating pregnancy, unspecified trimester | O99.519 |
| Hemoptysis | R04.2 |
| Cough | R05 |
| Acute cough | R05.1 |
| Subacute cough | R05.2 |
| Chronic cough | R05.3 |
| Cough syncope | R05.4 |
| Other specified cough | R05.8 |
| Cough, unspecified | R05.9 |
| Dyspnea | R06.0 |
| Dyspnea, unspecified | R06.00 |
| Orthopnea | R06.01 |
| Shortness of breath | R06.02 |
| Acute respiratory distress | R06.03 |
| Other forms of dyspnea | R06.09 |
| Stridor | R06.1 |
| Wheezing | R06.2 |
| Tachypnea, not elsewhere classified | R06.82 |
| Chest pain on breathing | R07.1 |
| Asphyxia and hypoxemia | R09.0 |
| Asphyxia | R09.01 |
| Hypoxemia | R09.02 |
| Respiratory arrest | R09.2 |
| Abnormal sputum | R09.3 |
| Nasal congestion | R09.81 |
| Other specified symptoms and signs involving the circulatory and respiratory systems | R09.89 |
| Fever of other and unknown origin* | R50 |
| Drug induced fever | R50.2 |
| Other specified fever | R50.8 |
| Fever presenting with conditions classified elsewhere | R50.81 |
| Postprocedural fever | R50.82 |
| Postvaccination fever | R50.83 |
| Febrile nonhemolytic transfusion reaction | R50.84 |
| Fever, unspecified | R50.9 |
| Shock, unspecified | R57.9 |
| Severe sepsis with septic shock | R65.21 |
| Chills (without fever) | R68.83 |

Supplementary Table 2. CVX, CPT, and NDC codes for influenza vaccines

| **Influenza Vaccine Type** | **CVX codes** | **CPT Codes** | **NDC Codes** |
| --- | --- | --- | --- |
| QIVe | 150, 158, 161, 166 | 90630,  90685, 90686, 90687, 90688 | 2017-2018: 19515-0896-01, 19515-0896-11, 19515-0912-41, 19515-0912-52, 33332-0317-01, 33332-0317-02, 33332-0417-10, 33332-0417-11, 49281-0712-40, 49281-0417-10, 49281-0417-50, 49281-0417-58, 49281-0417-88, 49281-0517-00, 49281-0517-25, 49281-0627-15, 49281-0627-78, 49281-0712-48, 58160-0907-41, 58160-0907-52  2018-2019: 19515-0900-01, 19515-0900-11, 19515-0909-41, 19515-0909-52, 33332-0318-01, 33332-0318-02, 33332-0418-10, 33332-0418-11, 49281-0418-00, 49281-0418-10, 49281-0418-50, 49281-0418-58, 49281-0418-88, 49281-0518-00, 49281-0518-25, 49281-0629-15, 49281-0629-78, 58160-0898-41, 58160-0898-52  2019-2020: 19515-0897-01, 19515-0897-11, 19515-0906-41, 19515-0906-52, 33332-0219-20, 33332-0219-21, 33332-0319-01, 33332-0319-02, 33332-0419-10, 33332-0419-11, 49281-0419-10, 49281-0419-50, 49281-0419-58, 49281-0419-88, 49281-0519-00, 49281-0519-25, 49281-0631-15, 49281-0631-78, 58160-0896-41, 58160-0896-52 |
| QIVc | 153, 171, 186 | 90661,  90674,  90756 | 2017-2018: 70461-0201-01, 70461-0201-11, 70461-0301-10, 70461-0301-12  2018-2019: 76420-0521-01, 70461-0318-03, 70461-0318-04, 70461-0418-10, 70461-0418-11  2019-2020: 70461-0319-03, 70461-0319-04, 70461-0419-11, 70461-0419-10 |

Abbreviations: CPT, Current Procedural Terminology; CVX, vaccine administered codes; NDC, National Drug Code; QIVc, cell-based quadrivalent influenza vaccine; QIVe, egg-based quadrivalent influenza vaccine.

Supplementary Table 3. Influenza test codes

| **Code type** | **Code** | **Long Name** | **Test Type** |
| --- | --- | --- | --- |
| CPT | 86710 | Antibody; influenza virus | Ab |
| CPT | 87275 | Infectious agent antigen detection by immunofluorescent technique; influenza B virus | Ag |
| CPT | 87276 | Infectious agent antigen detection by immunofluorescent technique; influenza A virus | Ag |
| CPT | 87400 | Infectious agent antigen detection by immunoassay technique, (e.g., enzyme immunoassay [EIA], enzyme-linked immunosorbent assay [ELISA], fluorescence immunoassay [FIA], immunochemiluminometric assay [IMCA]) qualitative or semiquantitative; Influenza, A or B, each | Ag |
| CPT | 87501 | Infectious agent detection by nucleic acid (DNA or RNA); influenza virus, includes reverse transcription, when performed, and amplified probe technique, each type or subtype | M |
| CPT | 87502 | Infectious agent detection by nucleic acid (DNA or RNA); influenza virus, for multiple types or sub- types, includes multiplex reverse transcription, when performed, and multiplex amplified probe technique, first 2 types or sub-types | M |
| CPT | 87503 | Infectious agent detection by nucleic acid (DNA or RNA); influenza virus, for multiple types or sub- types, includes multiplex reverse transcription, when performed, and multiplex amplified probe technique, each additional influenza virus type or sub-type beyond 2 (List separately in addition to code for primary procedure) | M |
| CPT | 87804 | Infectious agent antigen detection by immunoassay with direct optical observation; Influenza | Ag |
| LOINC | 100343-3 | Influenza virus B RNA [Presence] in Saliva (oral fluid) by NAA with probe detection | M |
| LOINC | 100344-1 | Influenza virus A RNA [Presence] in Saliva (oral fluid) by NAA with probe detection | M |
| LOINC | 15444-3 | Influenza virus A Ab [Presence] in Serum by Immune diffusion (ID) | Ab |
| LOINC | 20862-9 | Influenza virus A Ab [Presence] in Serum by Hemagglutination inhibition | Ab |
| LOINC | 22096-2 | Influenza virus A Ab [Presence] in Serum | Ab |
| LOINC | 22822-1 | Influenza virus A Ab [Presence] in Serum by Immunoassay | Ab |
| LOINC | 22825-4 | Influenza virus A Ag [Presence] in Specimen by Immune diffusion (ID) | Ag |
| LOINC | 24015-0 | Influenza virus A+B Ag [Presence] in Specimen | Ag |
| LOINC | 31858-4 | Influenza virus A Ag [Presence] in Throat | Ag |
| LOINC | 31859-2 | Influenza virus A Ag [Presence] in Specimen | Ag |
| LOINC | 31860-0 | Influenza virus A+B Ag [Presence] in Throat | Ag |
| LOINC | 31861-8 | Influenza virus A+B+C Ag [Presence] in Throat | Ag |
| LOINC | 31862-6 | Influenza virus A+B+C Ag [Presence] in Specimen | Ag |
| LOINC | 31863-4 | Influenza virus B Ag [Presence] in Throat | Ag |
| LOINC | 31864-2 | Influenza virus B Ag [Presence] in Specimen | Ag |
| LOINC | 33535-6 | Influenza virus A+B Ag [Presence] in Nasopharynx | Ag |
| LOINC | 34487-9 | Influenza virus A RNA [Presence] in Specimen by NAA with probe detection | M |
| LOINC | 38381-0 | Influenza virus A cDNA [Presence] in Specimen by NAA with probe detection | M |
| LOINC | 38382-8 | Influenza virus B [Presence] in Specimen by Organism specific culture | C |
| LOINC | 39311-6 | Influenza virus A N1 Ab [Presence] in Serum by Neutralization test | Ab |
| LOINC | 39312-4 | Influenza virus A N2 Ab [Presence] in Serum by Neutralization test | Ab |
| LOINC | 40981-3 | Deprecated Influenza virus A RNA [Presence] in Unspecified specimen by Probe & target amplification method | M |
| LOINC | 40982-1 | Influenza virus B RNA [Presence] in Specimen by NAA with probe detection | M |
| LOINC | 41141-3 | Influenza virus A N2 Ab [Presence] in Serum | Ab |
| LOINC | 41142-1 | Influenza virus A N1 Ab [Presence] in Serum | Ab |
| LOINC | 43837-4 | Influenza virus A IgG Ab [Presence] in Serum | Ab |
| LOINC | 43838-2 | Influenza virus A IgM Ab [Presence] in Serum | Ab |
| LOINC | 43839-0 | Influenza virus B IgG Ab [Presence] in Serum | Ab |
| LOINC | 43840-8 | Influenza virus B IgM Ab [Presence] in Serum | Ab |
| LOINC | 43851-5 | Influenza virus A IgA Ab [Presence] in Serum | Ab |
| LOINC | 43852-3 | Influenza virus B IgA Ab [Presence] in Serum | Ab |
| LOINC | 43874-7 | Influenza virus A Ag [Presence] in Nasopharynx | Ag |
| LOINC | 43895-2 | Influenza virus B Ag [Presence] in Nasopharynx | Ag |
| LOINC | 44556-9 | Influenza virus A Ab [Presence] in Serum by Complement fixation | Ab |
| LOINC | 44557-7 | Influenza virus A Ab [Presence] in Body fluid | Ab |
| LOINC | 44558-5 | Influenza virus A Ag [Presence] in Nasopharynx by Immunofluorescence | Ag |
| LOINC | 44559-3 | Influenza virus A Ag [Presence] in Bronchial specimen by Immunofluorescence | Ag |
| LOINC | 44560-1 | Influenza virus A Ag [Presence] in Nose by Immunofluorescence | Ag |
| LOINC | 44561-9 | Influenza virus A Ag [Presence] in Trachea by Immunofluorescence | Ag |
| LOINC | 44562-7 | Influenza virus A Ag [Presence] in Bronchial specimen | Ag |
| LOINC | 44563-5 | Influenza virus A Ag [Presence] in Nose | Ag |
| LOINC | 44564-3 | Influenza virus A Ag [Presence] in Nose by Immunoassay | Ag |
| LOINC | 44565-0 | Influenza virus A+B Ab [Presence] in Serum | Ab |
| LOINC | 44566-8 | Influenza virus A+B Ag [Presence] in Bronchial specimen | Ag |
| LOINC | 44567-6 | Influenza virus A+B Ag [Presence] in Nose | Ag |
| LOINC | 44568-4 | Influenza virus B Ab [Presence] in Serum by Complement fixation | Ab |
| LOINC | 44569-2 | Influenza virus B Ab [Presence] in Body fluid | Ab |
| LOINC | 44570-0 | Influenza virus B Ab [Presence] in Serum | Ab |
| LOINC | 44571-8 | Influenza virus B Ag [Presence] in Nasopharynx by Immunofluorescence | Ag |
| LOINC | 44572-6 | Influenza virus B Ag [Presence] in Bronchial specimen by Immunofluorescence | Ag |
| LOINC | 44573-4 | Influenza virus B Ag [Presence] in Nose by Immunofluorescence | Ag |
| LOINC | 44574-2 | Influenza virus B Ag [Presence] in Trachea by Immunofluorescence | Ag |
| LOINC | 44575-9 | Influenza virus B Ag [Presence] in Nose by Immunoassay | Ag |
| LOINC | 44576-7 | Influenza virus B Ag [Presence] in Bronchial specimen | Ag |
| LOINC | 44577-5 | Influenza virus B Ag [Presence] in Nose | Ag |
| LOINC | 44876-1 | Influenza virus A Ab [Presence] in Cerebral spinal fluid | Ab |
| LOINC | 45279-7 | Influenza virus A+B Ab [Presence] in Body fluid | Ab |
| LOINC | 46082-4 | Influenza virus A Ag [Presence] in Nasopharynx by Immunoassay | Ag |
| LOINC | 46083-2 | Influenza virus B Ag [Presence] in Nasopharynx by Immunoassay | Ag |
| LOINC | 47232-4 | Influenza virus A Ab [Presence] in Serum –1^st^ specimen | Ab |
| LOINC | 47233-2 | Influenza virus A Ab [Presence] in Serum –2^nd^ specimen | Ab |
| LOINC | 48310-7 | Influenza virus A [Presence] in Specimen by Organism specific culture | C |
| LOINC | 48509-4 | Influenza virus A and B RNA [Identifier] in Specimen by NAA with probe detection | M |
| LOINC | 49012-8 | Influenza virus A and B identified in Specimen by Bioassay | U |
| LOINC | 49520-0 | Influenza virus A H1 RNA [Presence] in Isolate by NAA with probe detection | M |
| LOINC | 49521-8 | Influenza virus A H1 RNA [Presence] in Specimen by NAA with probe detection | M |
| LOINC | 49522-6 | Influenza virus A H3 Ag [Presence] in Isolate by Immunofluorescence | Ag |
| LOINC | 49523-4 | Influenza virus A H3 RNA [Presence] in Isolate by NAA with probe detection | M |
| LOINC | 49524-2 | Influenza virus A H3 RNA [Presence] in Specimen by NAA with probe detection | M |
| LOINC | 49529-1 | Influenza virus A Ag [Presence] in Isolate by Immunofluorescence | Ag |
| LOINC | 49531-7 | Influenza virus A RNA [Presence] in Isolate by NAA with probe detection | M |
| LOINC | 49533-3 | Influenza virus B Ab [Presence] in Serum by Immunoassay | Ab |
| LOINC | 49534-1 | Influenza virus B Ag [Presence] in Isolate by Immunofluorescence | Ag |
| LOINC | 49535-8 | Influenza virus B RNA [Presence] in Isolate by NAA with probe detection | M |
| LOINC | 49537-4 | Influenza virus A and B RNA [Identifier] in Isolate by NAA with probe detection | M |
| LOINC | 49538-2 | Influenza virus identified in Specimen by Shell vial culture | C |
| LOINC | 50696-4 | Influenza virus A Ab [Identifier] in Serum by Neutralization test | Ab |
| LOINC | 50697-2 | Influenza virus A Ag [Identifier] in Isolate | Ag |
| LOINC | 50701-2 | Influenza virus A H1 Ag [Presence] in Isolate by Immunofluorescence | Ag |
| LOINC | 50707-9 | Influenza virus A polymerase B1 cDNA [Presence] in Isolate by Sequencing | M |
| LOINC | 53381-0 | Influenza virus A Ab [Identifier] in Serum | Ab |
| LOINC | 54240-7 | Influenza virus Ag [Presence] in Specimen | Ag |
| LOINC | 54241-5 | Influenza virus B Ag [Presence] in Isolate | Ag |
| LOINC | 54242-3 | Influenza virus identified in Isolate | U |
| LOINC | 54243-1 | Influenza virus RNA [Identifier] in Specimen by Probe | M |
| LOINC | 54244-9 | Influenza virus identified in Specimen | M |
| LOINC | 55465-9 | Influenza virus A H1 2009 pandemic RNA [Presence] in Specimen by NAA with probe detection | M |
| LOINC | 5860-2 | Influenza virus A Ag [Presence] in Throat by Immunoassay | Ag |
| LOINC | 5861-0 | Influenza virus A Ag [Presence] in Throat by Immunofluorescence | Ag |
| LOINC | 5862-8 | Influenza virus A Ag [Presence] in Specimen by Immunoassay | Ag |
| LOINC | 5863-6 | Influenza virus A Ag [Presence] in Specimen by Immunofluorescence | Ag |
| LOINC | 5864-4 | Influenza virus B Ag [Presence] in Throat by Immunoassay | Ag |
| LOINC | 5865-1 | Influenza virus B Ag [Presence] in Throat by Immunofluorescence | Ag |
| LOINC | 5866-9 | Influenza virus B Ag [Presence] in Specimen by Immunoassay | Ag |
| LOINC | 5867-7 | Influenza virus B Ag [Presence] in Specimen by Immunofluorescence | Ag |
| LOINC | 60538-6 | Influenza virus A H1+H3+B RNA [Presence] in Specimen by NAA with probe detection | M |
| LOINC | 61102-0 | Influenza virus A and B Ag [Identifier] in Specimen by Immunofluorescence | Ag |
| LOINC | 62462-7 | Influenza virus A+B RNA [Presence] in Specimen by NAA with probe detection | M |
| LOINC | 6435-2 | Influenza virus A+B Ag [Presence] in Throat by Immunoassay | Ag |
| LOINC | 6436-0 | Influenza virus A+B Ag [Presence] in Throat by Immunofluorescence | Ag |
| LOINC | 6437-8 | Influenza virus A+B Ag [Presence] in Specimen by Immunoassay | Ag |
| LOINC | 6438-6 | Influenza virus A+B Ag [Presence] in Specimen by Immunofluorescence | Ag |
| LOINC | 6439-4 | Influenza virus A+B+C Ag [Presence] in Throat by Immunoassay | Ag |
| LOINC | 6440-2 | Influenza virus A+B+C Ag [Presence] in Throat by Immunofluorescence | Ag |
| LOINC | 6441-0 | Influenza virus A+B+C Ag [Presence] in Specimen by Immunoassay | Ag |
| LOINC | 6442-8 | Influenza virus A+B+C Ag [Presence] in Specimen by Immunofluorescence | Ag |
| LOINC | 6601-9 | Influenza virus identified in Sputum by Organism specific culture | C |
| LOINC | 6602-7 | Influenza virus identified in Sputum tracheal aspirate by Organism specific culture | C |
| LOINC | 6603-5 | Influenza virus identified in Throat by Organism specific culture | C |
| LOINC | 6604-3 | Influenza virus identified in Specimen by Organism specific culture | C |
| LOINC | 72356-9 | Influenza virus A and B Ag [Identifier] in Specimen by Rapid immunoassay | Ag |
| LOINC | 72365-0 | Influenza virus A and B Ag [Identifier] in Nose by Immunofluorescence | Ag |
| LOINC | 72366-8 | Influenza virus A and B Ag [Identifier] in Nose by Rapid immunoassay | Ag |
| LOINC | 72367-6 | Influenza virus A+B Ag [Presence] in Nose by Rapid immunoassay | Ag |
| LOINC | 74784-0 | Influenza virus B lineage RNA [Identifier] in Specimen by NAA with probe detection | M |
| LOINC | 74785-7 | Influenza virus B Victoria lineage RNA [Presence] in Specimen by NAA with probe detection | M |
| LOINC | 74786-5 | Influenza virus B Yamagata lineage RNA [Presence] in Specimen by NAA with probe detection | M |
| LOINC | 76077-7 | Influenza virus A RNA [Presence] in Bronchoalveolar lavage by NAA with probe detection | M |
| LOINC | 76078-5 | Influenza virus A RNA [Presence] in Nasopharynx by NAA with probe detection | M |
| LOINC | 76079-3 | Influenza virus B RNA [Presence] in Bronchoalveolar lavage by NAA with probe detection | M |
| LOINC | 76080-1 | Influenza virus B RNA [Presence] in Nasopharynx by NAA with probe detection | M |
| LOINC | 77026-3 | Influenza virus A H1 RNA [Presence] in Nasopharynx by NAA with probe detection | M |
| LOINC | 77027-1 | Influenza virus A H3 RNA [Presence] in Nasopharynx by NAA with probe detection | M |
| LOINC | 77028-9 | Influenza virus A H1 2009 pandemic RNA [Presence] in Nasopharynx by NAA with probe detection | M |
| LOINC | 77383-8 | Influenza virus A Ag [Presence] in Bronchoalveolar lavage by Immunofluorescence | Ag |
| LOINC | 77384-6 | Influenza virus B Ag [Presence] in Bronchoalveolar lavage by Immunofluorescence | Ag |
| LOINC | 80204-1 | Influenza virus A and B identified in Nasopharynx by Shell vial culture | C |
| LOINC | 80382-5 | Influenza virus A Ag [Presence] in Upper respiratory specimen by Rapid immunoassay | Ag |
| LOINC | 80383-3 | Influenza virus B Ag [Presence] in Upper respiratory specimen by Rapid immunoassay | Ag |
| LOINC | 80588-7 | Influenza virus A M gene [Presence] in Nasopharynx by NAA with probe detection | M |
| LOINC | 80589-5 | Influenza virus A H1 HA gene [Presence] in Nasopharynx by NAA with probe detection | M |
| LOINC | 80590-3 | Influenza virus A H3 HA gene [Presence] in Nasopharynx by NAA with probe detection | M |
| LOINC | 80591-1 | Influenza virus B NS gene [Presence] in Nasopharynx by NAA with probe detection | M |
| LOINC | 82166-0 | Influenza virus A RNA [Presence] in Nasopharynx by NAA with non-probe detection | M |
| LOINC | 82167-8 | Influenza virus A H1 RNA [Presence] in Nasopharynx by NAA with non-probe detection | M |
| LOINC | 82168-6 | Influenza virus A H1 2009 pandemic RNA [Presence] in Nasopharynx by NAA with non-probe detection | M |
| LOINC | 82169-4 | Influenza virus A H3 RNA [Presence] in Nasopharynx by NAA with non-probe detection | M |
| LOINC | 82170-2 | Influenza virus B RNA [Presence] in Nasopharynx by NAA with non-probe detection | M |
| LOINC | 82461-5 | Influenza virus A and B and H1 2009 pandemic RNA [Identifier] in Upper respiratory specimen by NAA with probe detection | M |
| LOINC | 85477-8 | Influenza virus A RNA [Presence] in Upper respiratory specimen by NAA with probe detection | M |
| LOINC | 85478-6 | Influenza virus B RNA [Presence] in Upper respiratory specimen by NAA with probe detection | M |
| LOINC | 85821-7 | Influenza virus B Victoria lineage Ag [Presence] in Isolate by Hemagglutination inhibition | Ag |
| LOINC | 86318-3 | Influenza virus B Yamagata lineage Ag [Presence] in Isolate by Hemagglutination inhibition | Ag |
| LOINC | 86565-9 | Influenza virus A Ag [Presence] in Tissue by Immunofluorescence | Ag |
| LOINC | 86566-7 | Influenza virus A IgG Ab [Presence] in Serum by Immunoassay | Ab |
| LOINC | 86567-5 | Influenza virus A IgM Ab [Presence] in Serum by Immunoassay | Ab |
| LOINC | 86568-3 | Influenza virus A RNA [Presence] in Cerebral spinal fluid by NAA with probe detection | M |
| LOINC | 86569-1 | Influenza virus A RNA [Presence] in Tissue by NAA with probe detection | M |
| LOINC | 86570-9 | Influenza virus B IgM Ab [Presence] in Serum by Immunoassay | Ab |
| LOINC | 86571-7 | Influenza virus B RNA [Presence] in Cerebral spinal fluid by NAA with probe detection | M |
| LOINC | 86572-5 | Influenza virus B RNA [Presence] in Tissue by NAA with probe detection | M |
| LOINC | 88193-8 | Influenza virus A RNA [Presence] in Cornea or Conjunctiva by NAA with probe detection | M |
| LOINC | 88194-6 | Influenza virus B Ag [Presence] in Tissue by Immunofluorescence | Ag |
| LOINC | 88195-3 | Influenza virus B RNA [Presence] in Cornea or Conjunctiva by NAA with probe detection | M |
| LOINC | 88200-1 | Influenza virus identified in Cornea or Conjunctiva by Organism specific culture | C |
| LOINC | 88568-1 | Influenza virus identified in Lower respiratory specimen by Organism specific culture | C |
| LOINC | 88592-1 | Influenza virus B RNA [Presence] in Lower respiratory specimen by NAA with probe detection | M |
| LOINC | 88596-2 | Influenza virus B RNA [Presence] in Pericardial fluid by NAA with probe detection | M |
| LOINC | 88599-6 | Influenza virus A RNA [Presence] in Lower respiratory specimen by NAA with probe detection | M |
| LOINC | 88600-2 | Influenza virus A RNA [Presence] in Pericardial fluid by NAA with probe detection | M |
| LOINC | 88903-0 | Influenza virus B IgG Ab [Presence] in Serum by Immunoassay | Ab |
| LOINC | 88904-8 | Influenza virus A Ag [Presence] in Lower respiratory specimen by Immunofluorescence | Ag |
| LOINC | 88905-5 | Influenza virus B Ag [Presence] in Lower respiratory specimen by Immunofluorescence | Ag |
| LOINC | 91772-4 | Influenza virus identified in Upper respiratory specimen by Organism specific culture | C |
| LOINC | 92141-1 | Influenza virus B RNA [Presence] in Respiratory specimen by NAA with probe detection | M |
| LOINC | 92142-9 | Influenza virus A RNA [Presence] in Respiratory specimen by NAA with probe detection | M |
| LOINC | 92808-5 | Influenza virus A H3 RNA [Presence] in Upper respiratory specimen by NAA with probe detection | M |
| LOINC | 92809-3 | Influenza virus A H1 RNA [Presence] in Upper respiratory specimen by NAA with probe detection | M |
| LOINC | 92976-0 | Influenza virus B RNA [Presence] in Lower respiratory specimen by NAA with non-probe detection | M |
| LOINC | 92977-8 | Influenza virus A RNA [Presence] in Lower respiratory specimen by NAA with non-probe detection | M |
| LOINC | 94394-4 | Influenza virus A H3 RNA [Presence] in Lower respiratory specimen by NAA with probe detection | M |
| LOINC | 94395-1 | Influenza virus A H1 2009 pandemic RNA [Presence] in Lower respiratory specimen by NAA with probe detection | M |
| LOINC | 94396-9 | Influenza virus A H1 RNA [Presence] in Lower respiratory specimen by NAA with probe detection | M |
| LOINC | 99623-1 | Influenza virus A N1 RNA [Presence] in Specimen by NAA with probe detection | M |

Abbreviations: Ab, antibody; Ag, antigen; C, culture; LOINC; Logical Observation Identifiers Names and Codes; M, molecular detection (includes polymerase chain reaction); U, unknown type.

Supplementary Text 1. Case definitions

A case was defined by a positive influenza test result within the season timeframe ascertained using Current Procedural Terminology (CPT) or Logical Observation Identifiers Names and Codes (LOINC) (Supplementary Tables 1 and 2). Case definitions were explored and defined based on feasibility assessments preceding this study. During the feasibility assessment for this study, influenza test results were found to be reported in both structured and unstructured form in the data. Structured results refer to a Systematized Nomenclature of Medicine (SNOMED) concept. Unstructured, or non-standardized, results are short phrases sent from the lab or entered in the electronic health record (EHR) to describe the result. Influenza test results from the structured and unstructured data were classified as ‘Negative / Not Detected’ or ‘Positive / Detected’ as described below. Regarding the date of a test, if a test result had an available ordered date for the test, that date was used as the test date; if the ordered date was missing, the reported date of the test was used as the test date, and if that was missing, the date the record was created in the EHR was used as the test date.

If a patient had multiple test results in a given season, the following logic was implemented to select the relevant test result:

- If the patient had both positive and negative test results in a season, the only or earliest positive influenza test result was evaluated, and the patient was assigned as a case.
- Else if the patient had no negative influenza test results and more than one positive influenza test result in a season, the earliest positive influenza test result was evaluated, and the patient was assigned as a case.
- Else if the patient had no positive influenza test results and one or more negative results in a season, the only or earliest negative influenza test was evaluated, and the patient was assigned as a control.

Hence, each patient was assigned one status, either case or control, and controls had to have been tested negative through the end of the influenza season (i.e., have no positive results). This logic is consistent with the principle of cumulative seasonal incidence.

Supplementary Text 2. Mapping of structured and unstructured data to identify test results

Test results are reported in both structured and unstructured form. Structured results refer to a SNOMED concept and will be mapped to identify test results as shown in the table below.

| **SNOMED concept** | **SNOMED description** | **Assigned category** |
| --- | --- | --- |
| 260385009 | Negative | Negative / not detected |
| 260415000 | Not detected | Negative / not detected |
| 260373001 | Detected | Positive / detected |
| 10828004 | Positive | Positive / detected |

Abbreviation: SNOMED, Systematized Nomenclature of Medicine.

Unstructured, or non-standardized, results are short phrases sent from the lab or entered in the EHR to describe the result. The unstructured data are mapped to categories of ‘Negative/not detected’ and ‘Positive/detected’ for the purposes of this analysis using the following process:

1. First, entries that have both the terms ‘pos’ and ‘neg’ in them are mapped these to ‘Positive and Negative’ – for the purposes of these analyses, these terms will be considered Positive findings, as at least one flu type was marked to be positive.
   1. Using the results of the feasibility analysis, the most common unstructured values that are mapped to this term are: pos a / neg b, a- positive b- negative, neg a / pos b, a - positive b - negative. They comprise 0.4% of all results and 36.3% of all results mapped to ‘Positive and Negative’
2. Next, remaining entries that have either the term ‘neg’ or ‘not detected’ are mapped to ‘Negative/not detected’
   1. Using the results of the feasibility analysis, the most common unstructured values that are mapped to this term are: negative, not detected, neg, negative for influenza a and b. They comprise 55.5% of all results and 87.3% of all results mapped to ‘Negative/not detected’
3. Then, remaining entries that have either the term ‘pos’ or ‘detected’ are mapped to ‘Positive/detected’
   1. Using the results of the feasibility analysis, the most common unstructured values that are mapped to this term are: positive, detected, pos, positive a. They comprise 9.9% of all results and 83.2% of all results mapped to ‘Positive/detected’
4. Finally, the remaining entries are categorized as unmapped and cannot be used for analysis
   1. Using the results of the feasibility analysis, the most common unstructured values that are mapped to this term are: xyxyxy, pass, yes, present. They comprise 10.3% of all results and 65.4% of all unmapped non-numeric results.
   2. Numeric values from the unstructured data are also considered unmapped and not usable for this analysis. They comprise 7.5% of all results.

There are approximately 3.5k possible values in the Veradigm unstructured result field representing approximately 1.9M results, 1.6M of which were deemed to be usable as positive or negative. Note that some tests have multiple results, which is why the 1.6M relates to the smaller distinct test count with a usable result of 1.45M. The top 669 values, which represent 99% of all results, were reviewed manually to ensure that the mapping described above was accurate.

Supplementary Text 3. Statistical methodology

**Descriptive analysis**

For each influenza season, summary tables of patient baseline demographic and clinical characteristics were reported by vaccine type (QIVc, QIVe), by case status (influenza test positive vs influenza test negative), and by a cross between case status and vaccine type (cases with QIVc, controls with QIVc, cases with QIVe, controls with QIVe). Categorical variables were presented as counts and percentages, continuous variables were presented by mean, SD, median, and IQR. The proportion of patients with missing or unknown, or undocumented values for race and ethnicity were also reported. The percentage of patients with test dates in each week of the influenza season was summarized by vaccine type and by case status in descriptive tables and presented as figures. Vaccine exposure group balance for covariates among the controls and univariate comparisons between positive cases and negative controls were assessed using Austin’s SMD [2].

**Covariate balance—standardized mean differences**

In a test-negative design study, the control group is intended to represent the overall source population (i.e., the population vaccinated with QIVc or QIVe). It is within this control group that characteristics of patients in each vaccine group are evaluated for potential confounding [3]. For this study, covariate balance between vaccine exposure group among the controls was assessed using standardized mean differences (SMDs). An SMD value with an absolute value ≤ 0.1 was used to indicate a negligible difference in proportions between the groups [2]. SMDs were generated to assess differences in each variable between the QIVc and QIVe controls prior to any statistical adjustment or weighting. SMDs were also generated to assess univariate differences in each variable between cases and controls.

The SMD compares the difference in means in units of the pooled standard deviation. Unlike t-tests and other statistical tests of hypothesis, the SMD is not influenced by sample size. Thus, the use of the SMD can be used to compare the balance in measured variables between the QIVc- and QIVe-exposed controls in the unweighted sample with balance in a weighted sample.

For continuous variables, the SMD is defined as:

$$d= \frac{(\bar{x}_{group 1}- \bar{x}_{group 0})}{\sqrt{\frac{s_{group 1}^{2}+ s_{group 0}^{2}}{2}}}$$

where $\bar{x}_{group 1}$ and $\bar{x}_{group 0}$ denote the sample means of the covariate in the QIVc and QIVe exposure groups among controls (or case and control group for the univariate comparison), while $s_{group 1}^{2}$and $s_{group 0}^{2}$ denote the sample variances of the covariate in the QIVc and QIVe group (or case and control group).

For categorical variables, the SMD is defined as:

$$d= \frac{(\hat{p}_{group 1-}\hat{p}_{group 0})}{\sqrt{\frac{\hat{p}_{group 1}\left( 1- \hat{p}_{group 1} \right)+ \hat{p}_{group 0}\left( 1- \hat{p}_{group 0} \right)}{2}}}$$

where $\hat{p}_{group 1}$ and $\hat{p}_{group 0}$ denote the prevalence or means of the categorical variable in the QIVc and QIVe exposure group among the controls (or case and control group).

**Primary analyses**

The rVE between QIVc and QIVe was assessed using a retrospective test-negative design. With this design, the same criteria for clinical presentation is used for selection of both cases and controls, minimizing the likelihood for selection bias [4, 5]. This principle was applied in the current study by restricting to vaccinated patients with a documented ARFI within 7 days (before or after) of the influenza test during the influenza season.

The Primary Analyses comprised a series of generalized linear models (Supplementary Table 4) with a logit link to obtain ORs comparing influenza-positive cases and influenza-negative controls, with vaccine type received as the exposure of interest. The rVEs were estimated by comparing the odds of testing positive for influenza among QIVc recipients with the odds among QIVe recipients and were calculated using the formula rVE = (1-OR)*100% and reported with 95% confidence intervals. The main model used a doubly robust approach combining multivariable adjustment with IPTW of the population. For transparency, a stepwise implementation of the main model was applied. First, an unadjusted rVE was calculated using a model with vaccine type only (Model 1). Model 2 used an IPTW approach that created a weighted sample that produced a weighted rVE. Model 3 included vaccine type and covariates to produce a multivariable model adjusted rVE (using the unweighted patient population). Finally, Model 4, the main analysis, employed a doubly robust approach that combined weighting and adjusting such that ORs and the main adjusted rVE were estimated in an IPTW-weighted sample using a multivariable regression model.

**Spline of test date**

All analyses, except for the sensitivity analysis matched on test week, adjusted for calendar time between the start of the influenza season and the test date to account for potential waning of vaccine effectiveness as the season progresses and variation in the risk of infection and strain distribution over time. Because it is not assumed that elapsed time has a linear effect, the variable for time from start of the influenza season to test date was transformed using a spline function to summarize the non-linear relationship between elapsed time and influenza test result [6]. The approach used a restricted (natural) cubic spline to split the range of values of elapsed time into intervals using ‘knots’ that defined where one interval ended and another began. Prior to inclusion in the adjusted analysis, the number (e.g., 3, 4, or 5) and placement (e.g., at quartiles, quintiles, etc.) of knots were examined and the PSPLINET SAS macro was used to visualize how the spline function parsed the data for the examined number of knots [7]. Ideal placement and number of knots was informed by best practice and the Akaike information criteria assessing goodness of fit [8]. Splines used in the analysis had 5 knots located at quintiles using the MAKESPL SAS macro [9].

**Weighting using inverse probability treatment weighting**

For Model 2 and the main analysis (Model 4), stabilized IPTWs were used to account for treatment effects adjusted for age (continuous), sex, region, test date (as spline), and any additional covariate with SMD > 0.1, excluding test type. IPTW allowed maximal use of available data but may be distorted by cases with very large or very small propensity scores, which may result in very small or large IPTW values. Stabilized weighting avoids the effect of extreme weights on the variability of the estimated treatment effect.

The methodology used to perform stabilized IPTW is as follows:

1. Among the influenza-test-negative controls, which represent the overall source population, separate regression models for each season predictive of treatment group membership (i.e., QIVc versus QIVe) provided propensity scores (pj) based on the study covariates. The models included a priori covariates and additional covariates identified as has having a non-negligible difference (unbalanced) between vaccine groups among the controls (|SMD| >0.1)
2. These fitted models were then applied to the influenza-positive cases to calculate propensity of treatment group membership scores for the cases (pj).
3. Next, both cases and controls in each of the vaccine groups were assigned a stabilized weight as follows:
   1. IPTW values wj were calculated as wj = 1 / (pj) for the QIVc group and wj = 1 / (1-pj) for the QIVe group.
   2. Stabilized weights w*j were obtained by multiplying IPTW values by the proportion of individuals in the QIVc and QIVe groups, respectively. For the QIVc group w*j = pt * wj, while for the QIVc group w*j = (1- pt) * wj where pt = Nt / (Nt+Nc), the proportion of individuals in the QIVc group.
4. Comparability between the unweighted and weighted vaccine groups was assessed by comparing distributions of variables included in the IPTW weighting.
5. SMDs were calculated for all covariates and absolute standardized differences in the unweighted and weighted covariates between QIVc and QIVe were displayed graphically.

| Estimate π=Pr(outcome of interest=1\|x) for logit(π)= α + β’x  where x is a single item vector, vaccine type and each observation is appropriately IPTW weighted for the vaccine pair being compared (QIVc:QIVe) |
| --- |

**Adjusting using multivariable modelling**

For Model 3, an OR comparing influenza-positive cases and influenza-negative controls were estimated using a multivariable regression model that included vaccine type as the exposure and a priori covariates – age, sex, region, test date (as spline) – and any additional covariate with SMD > 0.1, excluding test type.

**Doubly robust main analysis**

For Model 4, the main analysis, a doubly robust approach was used to further adjust for any potential residual confounding [10]. The IPTW sample was used in a multivariable model with age, sex, region, test date (as spline), and any other covariates that remain unbalanced following IPTW, excluding test type.

| Estimate π=Pr(influenza positive=1\|x) for logit(π)= α + β’x  where each observation is appropriately IPTW weighted and x is a multi-item vector of vaccine type (QIVc versus QIVe), a priori defined covariates and remaining unbalanced study covariates |
| --- |

**Sensitivity analyses**

Sensitivity analyses were conducted to quantify the robustness of the main results to possible influences of unmeasured confounding, measurement error, and selection biases. A summary of the sensitivity analysis can be found in (Supplementary Table 4).

*Propensity to be tested*

This sensitivity analysis addressed the potential bias of which patients are given an influenza test. The analysis included all vaccinated (with QIVc or QIVe) patients regardless of testing status with a documented ARFI during the influenza season, for whom we calculated the propensity to be tested during the influenza season. Demographic (age, sex, race, ethnicity, and region) and clinical (CCI score, high-risk conditions, healthcare utilization, and week of vaccination) covariates were used to estimate the propensity to be tested and propensity-for-testing estimators were created using the inverse of the score. The main analysis (Model 4) was then repeated, accounting for the propensity-for-testing estimator as an additional covariate in the model.

*Peak season*

This sensitivity analysis limited to the subset of the population in the main analysis whose test date fell within the peak influenza season to examine potential bias (underestimation of rVE) from lower predictive ability of rapid antigen tests when influenza activity is low. Limiting test dates within a period of higher test positivity decreases the possibility of case misclassification among rapid tests during periods of low influenza activity [11, 12].

For the 2017–2018 season, peak season was defined as the time period from December 11, 2017 to March 18, 2018. For the 2018–2019 season, peak season was defined as the time period from December 17, 2018 to April 7, 2019. For the 2019–2020 season, peak season was defined as the time period from December 8, 2019 to March 7, 2019. The peak season cut points were determined using the moving epidemic method (MEM), based on CDC data on the percentage of outpatient influenza tests that were positive [13–15]. The MEM – a standard approach to establishing epidemic thresholds using historical epidemiological data – is widely applied in Europe and has also been used in recent research in the U.S. assessing the rVE of influenza vaccines [16].

For this sensitivity analysis, the sample was re-weighted using IPTW and the model was re-specified, following the same procedure described for the primary analysis.

*Matching on week of test date*

In this sensitivity analysis, rather than adjusting for time from influenza season start date to test date using a spline, cases were matched to all available test-negative controls on the week of the test week. The intent was to further examine if there was confounding associated with the timing of the test via a matched analysis, since this is less sensitive to model misspecification. A greedy nearest neighbor approach was used to match cases to controls by test week only. Case to control ratios of 1:1, 1:2, and 1:3 were explored. Based on the number of cases retained, for the 2017–2018 season the matched analysis included a 1:2 case to control match. For the 2018–2019 and 2019–2020 seasons, matching was 1:1. The subset of patients used in this analysis will be referred to as the Matched on Week Population. After matching on calendar week, the sample was re-weighted in all matched patients using IPTW and Model 4 was re-specified excluding the spline function for test date as a covariate.

*Exclusion of antibody and culture tests*

This sensitivity analysis was limited to the subset of the population in the main analysis whose test type was antigen or molecular to address potential bias due to lack of specificity of antibody tests to reliably detect acute disease and potential differences in patients tested using culture methods.

For this sensitivity analysis, the sample was re-weighted using IPTW and the model was re-specified, following the same procedure described for the primary analysis. Compared with the primary analysis, when limiting to antigen and molecular tests, a few additional covariates in each season were imbalanced prior to weighting. In addition to the variables included in the primary analysis IPTW, the reweighting also included obesity in all three seasons; chronic lung disease and weakened immune system in the 2018-2019 and 2019-2020 seasons; race, ethnicity, and Charlson Comorbidity Index (CCI) score in the 2017-2018 season; and liver disease in the 2019-2020 season. After weighting, all covariates were balanced.

**Exploratory sensitivity analysis:** **test sensitivity and specificity**

Because the number of cases for each test type was too low to perform a subgroup analysis, we simulated the potential impact of test type to account for potential misclassification associated with the sensitivity and specificity of the test type received. Antigen tests for influenza are commonly used but have a lower sensitivity than molecular detection-based tests [17], potentially leading to greater misclassification of case status. This analysis was restricted to patients tested with either molecular detection-based tests (including PCR) or antigen tests, as antibody tests and culture tests are estimated to account for a small percentage of the study population and the primary concern is the potential for misclassification among antigen relative to molecular detection-based tests.

Pre-specified sensitivity and specificity parameter ranges for antigen tests and for molecular detection-based tests (see table below) were incorporated in a quantitative simulation that corrected for potential misclassification of case status accounting for test-specific sensitivity and specificity through probabilistic Monte Carlo simulation methods following guidelines and a programming macro developed to assess systematic errors in observational data [18, 19]. This method simulates the data that would have been observed had the misclassified case status been correctly classified given the sensitivity and specificity of the influenza tests.

**Assumed Average Sensitivity and Specificity Parameters for Antigen and Molecular Tests**

|  | **95% CI** | **Antigen** | **Molecular** |
| --- | --- | --- | --- |
| **Sensitivity** | Lower limit | .821 | .96 |
|  | Upper limit | .955 | 1 |
| **Specificity** | Lower limit | .901 | .94 |
|  | Upper limit | .942 | 1 |

Assumed values for antigen tests were calculated from an average of sensitivity or specificity estimates for three FDA-cleared and Clinical Laboratory Improvement Amendments waived antigen tests [17, 20–22]. Assumed values for molecular tests were obtained from a recent prospective study on the accuracy of a rapid reverse transcription PCR assay for point-of-care detection of influenza A/B virus at emergency department admission during the 2017–2018 influenza season [23].

Following guidance from the literature [18], a trapezoidal probability density function was specified, which specifies a lower and upper mode in addition to the lower and upper bounds (here, 95% CIs as listed in above table). For each iteration of the macro, a value of sensitivity and specificity was selected from the probability density function. Then, using the number of observed test-positive cases and test-negative controls from the study population and the selected value for sensitivity and specificity, the number of expected cases and controls was determined to calculate a positive predictive value and negative predictive value, conditional on vaccine type, representing the probability that those originally classified as cases were correctly classified and the probability that those originally classified as controls were correctly classified, respectively. That is, the predictive values were used as the probability that each individual was correctly classified, conditional on their original exposure and case-control status. Next, a random number was chosen between 0 and 1 and compared to the relevant predictive value for each individual. If the random number was greater than the predicative value, the individual’s case status was reclassified. In this manner, a reconstructed dataset was created with case status accounting for test-specific sensitivity and specificity.

The macro outputs an OR from the reconstructed dataset, representing one possible summary measure of association that could have occurred after accounting for misclassification of case status. The macro was programmed to run for 3,000 iterations; the number of iterations was selected to balance the number of simulations with required computing time. By setting the macro to run for multiple iterations, the macro creates a distribution of ORs accounting for systematic error. The macro then also applies a correction for random error, producing a 95% confidence interval that accounts for both the systematic and random error.

Because the macro does not allow for IPTW, an adjusted rVE estimate was then recalculated accounting for potential misclassification of case status using the parameters specified in the multivariable model (Model 3) from the primary analysis. To support comparison, the rVE from the multivariable Model 3 was also calculated in a population similarly restricted to molecular and antigen tests.

**Post-hoc analyses**

*Multivariable regression using matched on week population*

The first post-hoc analysis assessed the rVE using the multivariable regression model (Model 3) from the primary analysis for the matched on week population. This matched sample was created for the sensitivity analysis that matched cases to all available controls on the week of the index test date. A greedy nearest neighbor approach was used to match cases to controls by test week only. Case to control ratios of 1:1, 1:2, and 1:3 were explored. Based on the number of cases retained, for the 2017–2018 season, the matched analysis included a 1:2 case to control match. For the 2018–2019 and 2019–2020 seasons, matching was 1:1.

*Peak season analysis of matched sample*

A second post-hoc analysis replicated the initial post-hoc multiple regression analysis but further restricted to the peak influenza season. The peak season was defined consistent with the per-protocol peak season sensitivity analysis. For the 2017–2018 season, peak season was defined as the time period from December 11, 2017 to March 18, 2018. For the 2018–2019 season, peak season was defined as the time period from December 17, 2018 to April 7, 2019. For the 2019–2020 season, peak season was defined as the time period from December 8, 2019 to March 7, 2019.

**Missing values**

Among demographic variables, missing values were captured as a category—i.e., as “Missing” or “Not Reported.” Missing values were not imputed. Patients with missing values for age or sex were excluded from the study.

For the continuous variable CCI, patients were assigned a score of 0 if they had no record of any of the conditions used for the CCI index.

Supplementary Table 4. Summary of statistical analyses

| **Analysis** | **Description** | **Weighting variables** | **Multivariable model covariates** |
| --- | --- | --- | --- |
| **Descriptive analysis** | | | |
| 1. Patient Demographic and Clinical Characteristics | Descriptive statistics for covariates by case status, vaccine type, and case status by vaccine type; SMDs | N/A | N/A |
| **Primary analyses** | | | |
| 1. Unadjusted rVEs and 95% CIs | rVE, unweighted, no covariates | N/A | N/A |
| 2. rVEs and 95% CIs weighted using IPTW | rVE, weighted | Age (continuous), sex, region, test date (as spline), and any other covariate with \|SMD\| > 0.1 | N/A |
| 3. rVEs and 95% CIs adjusted using multivariable modeling | rVE, covariate adjusted | N/A | Age (continuous), sex, region, test date (as spline), and any other covariate with \|SMD\| > 0.1 |
| **4. Main analysis: rVEs and 95% CIs adjusted using a ‘doubly robust’ approach: IPTW and multivariable modelling** | **rVE, weighted, covariate adjusted** | **Age (continuous), sex, region, test date (as spline), and any other covariate with \|SMD\| > 0.1** | **Age (continuous), sex, region, test date (as spline), and any other covariate that remains unbalanced after weighting, i.e., weighted \|SMD\| > 0.1** |
| **Sensitivity analyses** | | | |
| 1. Propensity-to-be-tested sensitivity analysis | rVE, weighted, covariate adjusted | Age (continuous), sex, region, test date (as spline), and any other covariate with \|SMD\| > 0.1 | Age (continuous), sex, region, test date (as spline), propensity-to-be-tested estimator, and any other covariate that remains unbalanced after weighting, i.e., weighted \|SMD\| > 0.1 |
| 2. Matching on Test Week sensitivity analysis | rVE, weighted, covariate adjusted | Age (continuous), sex, region, and any other covariate with \|SMD\| > 0.1 | Age (continuous), sex, region, and any other covariate that remains unbalanced after weighting, i.e., weighted \|SMD\| > 0.1 |
| 3. Peak influenza season sensitivity analysis | rVE, weighted, covariate adjusted | Age (continuous), sex, region, test date (as spline), and any other covariate with \|SMD\| > 0.1 | Age (continuous), sex, region, test date (as spline), and any other covariate that remains unbalanced after weighting, i.e., weighted \|SMD\| > 0.1 |
| 4. Exclusion of antibody and culture tests | rVE, weighted, covariate adjusted | Age (continuous), sex, region, test date (as spline), and any other covariate with \|SMD\| > 0.1 | Age (continuous), sex, region, test date (as spline), and any other covariate that remains unbalanced after weighting, i.e., weighted \|SMD\| > 0.1 |
| **Exploratory sensitivity analysis** | | | |
| 1. Test sensitivity and specificity | rVE, covariate adjusted, case status misclassification correction | N/A | Age (continuous), sex, region, test date (as spline), and any other covariate with \|SMD\| > 0.1 |
| **Post-hoc analyses (2019–2020 season only)** | | | |
| 1. rVEs and 95% CIs adjusted using multivariable model, test week-matched sample | rVE, covariate adjusted | N/A | Age (continuous), sex, region, and any other covariate with \|SMD\| > 0.1 |
| 2. rVEs and 95% CIs adjusted using multivariable model, test week-matched sample, limited to peak influenza season | rVE, covariate adjusted | N/A | Age (continuous), sex, region, and any other covariate with \|SMD\| > 0.1 |

Abbreviations: CI, confidence interval; IPTW, inverse probability of treatment weighting; N/A, not applicable; QIVc, cell-based quadrivalent influenza vaccine; QIVe, egg-based quadrivalent influenza vaccine; rVE, relative vaccine effectiveness; SMD, standardized mean difference.

Supplementary Table 5. Final covariates in adjusted statistical models of the primary analyses

| **Season** | **Model 2: IPTW** | **Model 3: multivariable regression** | **Model 4: main analysis: doubly robust**  **(IPTW and multivariable)** |
| --- | --- | --- | --- |
| 2017–2018 | A priori: Age, Sex, Region, test date spline  Based on SMD (>\|0.1): Neurologic disorder, Blood disorder, Endocrine disorder, Heart disease, Metabolic disorder | A priori: Age, Sex, Region, test date spline  Based on SMD (>\|0.1): Neurologic disorder, Blood disorder, Endocrine disorder, Heart disease, Metabolic disorder | IPT weight  A priori: Age, Sex, Region, test date spline  Based on SMD: Race |
| 2018–2019 | A priori: Age, Sex, Region, test date spline  Based on SMD: CCI Score, Liver disease, Neurologic disorder, Blood disorder, Endocrine disorder, Heart disease, Metabolic disorder | A priori: Age, Sex, Region, test date spline  Based on SMD: CCI Score, Liver disease, Neurologic disorder, Blood disorder, Endocrine disorder, Heart disease, Metabolic disorder | IPT weight  A priori: Age, Sex, Region, test date spline  Based on SMD: None |
| 2019–2020 | A priori: Age, Sex, Region, test date spline  Based on SMD: CCI Score, Neurologic disorder, Blood disorder, Endocrine disorder, Heart disease, Metabolic disorder | A priori: Age, Sex, Region, test date spline   Based on SMD: CCI Score, Neurologic disorder, Blood disorder, Endocrine disorder, Heart disease, Metabolic disorder | IPT weight  A priori: Age, Sex, Region, test date spline  Based on SMD: None |

Abbreviations: CCI, Charlson comorbidity index; IPTW, inverse probability of treatment weighting; SMD, standardized mean difference.

Supplementary Table 6. Demographic and clinical characteristics of the study population at index date, irrespective of case status

| **Characteristic** | **2017–2018** | | | **2018–2019** | | | **2019–2020** | | |
| --- | --- | --- | --- | --- | --- | --- | --- | --- | --- |
|  | **All (N=31,824)** | **QIVc (n=3,115)** | **QIVe (n=28,709)** | **All (N=33,388)** | **QIVc (n=3,426)** | **QIVe (n=29,962)** | **All (N=34,398)** | **QIVc (n=3,890)** | **QIVe (n=30,508)** |
| Age, mean (SD) | 29.1 (21.5) | 42.5 (17.6) | 27.6 (21.4) | 24.9 (20.3) | 38.8 (18.5) | 23.3 (19.9) | 23.6 (19.6) | 37.2 (18.6) | 21.8 (19.1) |
| Age group, n (%) |  |  |  |  |  |  |  |  |  |
| 4-17 years | 15,000 (47.1) | 436 (14.0) | 14,564 (50.7) | 18,611 (55.7) | 700 (20.4) | 17,911 (59.8) | 19,903 (57.9) | 865 (22.2) | 19,038 (62.4) |
| 18-49 years | 8,166 (25.7) | 1,257 (40.4) | 6,909 (24.1) | 8,276 (24.8) | 1,436 (41.9) | 6,840 (22.8) | 8,642 (25.1) | 1,735 (44.6) | 6,907 (22.6) |
| 50-64 years | 8,658 (27.2) | 1,422 (45.7) | 7,236 (25.2) | 6,501 (19.5) | 1,290 (37.7) | 5,211 (17.4) | 5,853 (17.0) | 1,290 (33.2) | 4,563 (15.0) |
| Sex, n (%) |  |  |  |  |  |  |  |  |  |
| Female | 19,080 (60.0) | 2,045 (65.7) | 17,035 (59.3) | 19,631 (58.8) | 2,228 (65.0) | 17,403 (58.1) | 20,119 (58.5) | 2,521 (64.8) | 17,598 (57.7) |
| Male | 12,744 (40.0) | 1,070 (34.3) | 11,674 (40.7) | 13,757 (41.2) | 1,198 (35.0) | 12,559 (41.9) | 14,279 (41.5) | 1,369 (35.2) | 12,910 (42.3) |
| Race, n (%) |  |  |  |  |  |  |  |  |  |
| White | 21,672 (68.1) | 2,257 (72.5) | 19,415 (67.6) | 22,697 (68.0) | 2,431 (71.0) | 20,266 (67.6) | 23,578 (68.5) | 2,676 (68.8) | 20,902 (68.5) |
| Black | 2,093 (6.6) | 207 (6.6) | 1,886 (6.6) | 2,200 (6.6) | 171 (5.0) | 2,029 (6.8) | 1,913 (5.6) | 186 (4.8) | 1,727 (5.7) |
| Asian | 634 (2.0) | 51 (1.6) | 583 (2.0) | 734 (2.2) | 88 (2.6) | 646 (2.2) | 733 (2.1) | 111 (2.9) | 622 (2.0) |
| Other | 729 (2.3) | 30 (1.0) | 699 (2.4) | 816 (2.4) | 41 (1.2) | 775 (2.6) | 839 (2.4) | 64 (1.6) | 775 (2.5) |
| Unknown/not reported | 6,696 (21.0) | 570 (18.3) | 6,126 (21.3) | 6,941 (20.8) | 695 (20.3) | 6,246 (20.8) | 7,335 (21.3) | 853 (21.9) | 6,482 (21.2) |
| Ethnicity, n (%) |  |  |  |  |  |  |  |  |  |
| Hispanic | 2,222 (7.0) | 148 (4.8) | 2,074 (7.2) | 2,926 (8.8) | 217 (6.3) | 2,709 (9.0) | 2,932 (8.5) | 283 (7.3) | 2,649 (8.7) |
| Non-Hispanic | 26,942 (84.7) | 2,757 (88.5) | 24,185 (84.2) | 27,441 (82.2) | 2,871 (83.8) | 24,570 (82.0) | 27,991 (81.4) | 3,189 (82.0) | 24,802 (81.3) |
| Unknown/not reported | 2,660 (8.4) | 210 (6.7) | 2,450 (8.5) | 3,021 (9.0) | 338 (9.9) | 2,683 (9.0) | 3,475 (10.1) | 418 (10.7) | 3,057 (10.0) |
| Geographic region, n (%) |  |  |  |  |  |  |  |  |  |
| Northeast | 3,284 (10.3) | 179 (5.7) | 3,105 (10.8) | 3,582 (10.7) | 210 (6.1) | 3,372 (11.3) | 5,155 (15.0) | 269 (6.9) | 4,886 (16.0) |
| Midwest | 9,655 (30.3) | 413 (13.3) | 9,242 (32.2) | 6,671 (20.0) | 258 (7.5) | 6,413 (21.4) | 6,981 (20.3) | 407 (10.5) | 6,574 (21.5) |
| South | 17,744 (55.8) | 2,426 (77.9) | 15,318 (53.4) | 21,336 (63.9) | 2,758 (80.5) | 18,578 (62.0) | 20,116 (58.5) | 2,783 (71.5) | 17,333 (56.8) |
| West | 1,141 (3.6) | 97 (3.1) | 1,044 (3.6) | 1,799 (5.4) | 200 (5.8) | 1,599 (5.3) | 2,146 (6.2) | 431 (11.1) | 1,715 (5.6) |
| Influenza test type, n (%)^a^ |  |  |  |  |  |  |  |  |  |
| Antigen | 29,619 (93.1) | 2,992 (96.1) | 26,627 (92.7) | 30,862 (92.4) | 3,095 (90.3) | 27,767 (92.7) | 27,955 (81.3) | 3,093 (79.5) | 24,862 (81.5) |
| Molecular | 1,801 (5.7) | 112 (3.6) | 1,689 (5.9) | 2,038 (6.1) | 314 (9.2) | 1,724 (5.8) | 6,095 (17.8) | 677 (17.4) | 5,418 (17.7) |
| Culture | 264 (0.8) | 5 (0.2) | 259 (0.9) | 293 (0.9) | 7 (0.2) | 286 (1.0) | 70 (0.2) | 2 (0.1) | 68 (0.2) |
| Antibody | 140 (0.4) | 6 (0.2) | 134 (0.5) | 195 (0.6) | 10 (0.3) | 185 (0.6) | 278 (0.8) | 118 (3.0) | 160 (0.5) |
| CCI, mean (SD) | 0.5 (1.1) | 0.6 (1.2) | 0.5 (1.0) | 0.4 (0.9) | 0.5 (1.1) | 0.4 (0.9) | 0.4 (0.9) | 0.5 (1.0) | 0.3 (0.8) |
| High-risk conditions, n (%) |  |  |  |  |  |  |  |  |  |
| ≥1 High-risk condition | 17,719 (55.7) | 2,174 (69.8) | 15,545 (54.1) | 16,451 (49.3) | 2,190 (63.9) | 14,261 (47.6) | 16,403 (47.7) | 2,371 (61.0) | 14,032 (46.0) |
| Heart disease and related conditions | 8,926 (28.0) | 1,363 (43.8) | 7,563 (26.3) | 7,095 (21.3) | 1,253 (36.6) | 5,842 (19.5) | 6,520 (19.0) | 1,266 (32.5) | 5,254 (17.2) |
| Metabolic disorders | 8,132 (25.6) | 1,315 (42.2) | 6,817 (23.7) | 6,649 (19.9) | 1,191 (34.8) | 5,458 (18.2) | 6,243 (18.1) | 1,188 (30.5) | 5,055 (16.6) |
| Endocrine disorders | 6,423 (20.2) | 980 (31.5) | 5,443 (19.0) | 5,214 (15.6) | 959 (28.0) | 4,255 (14.2) | 4,864 (14.1) | 935 (24.0) | 3,929 (12.9) |
| Asthma | 5,440 (17.1) | 453 (14.5) | 4,987 (17.4) | 5,431 (16.3) | 516 (15.1) | 4,915 (16.4) | 5,574 (16.2) | 590 (15.2) | 4,984 (16.3) |
| Blood disorders | 3,232 (10.2) | 452 (14.5) | 2,780 (9.7) | 2,893 (8.7) | 472 (13.8) | 2,421 (8.1) | 2,813 (8.2) | 469 (12.1) | 2,344 (7.7) |
| Obesity (BMI ≥40 kg/m^2^) | 2,656 (8.3) | 362 (11.6) | 2,294 (8.0) | 2,327 (7.0) | 354 (10.3) | 1,973 (6.6) | 2,267 (6.6) | 380 (9.8) | 1,887 (6.2) |
| Weakened immune system | 1,629 (5.1) | 233 (7.5) | 1,396 (4.9) | 1,341 (4.0) | 222 (6.5) | 1,119 (3.7) | 1,312 (3.8) | 241 (6.2) | 1,071 (3.5) |
| Neurologic and neurodevelopmental conditions | 1,473 (4.6) | 42 (1.3) | 1,431 (5.0) | 1,779 (5.3) | 49 (1.4) | 1,730 (5.8) | 2,068 (6.0) | 94 (2.4) | 1,974 (6.5) |
| Chronic lung disease | 1,447 (4.5) | 184 (5.9) | 1,263 (4.4) | 1,087 (3.3) | 186 (5.4) | 901 (3.0) | 953 (2.8) | 183 (4.7) | 770 (2.5) |
| Liver disorders | 987 (3.1) | 150 (4.8) | 837 (2.9) | 818 (2.4) | 169 (4.9) | 649 (2.2) | 774 (2.3) | 156 (4.0) | 618 (2.0) |
| Kidney diseases | 737 (2.3) | 115 (3.7) | 622 (2.2) | 476 (1.4) | 87 (2.5) | 389 (1.3) | 441 (1.3) | 81 (2.1) | 360 (1.2) |
| Stroke | 417 (1.3) | 71 (2.3) | 346 (1.2) | 321 (1.0) | 55 (1.6) | 266 (0.9) | 281 (0.8) | 50 (1.3) | 231 (0.8) |
| Baseline all-cause healthcare resource utilization |  |  |  |  |  |  |  |  |  |
| Outpatient visits, mean (SD) | 5.7 (5.6) | 5.3 (5.9) | 5.7 (5.5) | 5.4 (5.2) | 5.3 (5.4) | 5.4 (5.2) | 5.3 (5.2) | 5.6 (5.8) | 5.3 (5.1) |
| Inpatient admissions, n (%) |  |  |  |  |  |  |  |  |  |
| 0 | 29,760 (93.5) | 2,858 (91.7) | 26,902 (93.7) | 31,588 (94.6) | 3,183 (92.9) | 28,405 (94.8) | 32,662 (95.0) | 3,652 (93.9) | 29,010 (95.1) |
| 1 | 1,340 (4.2) | 168 (5.4) | 1,172 (4.1) | 1,279 (3.8) | 167 (4.9) | 1,112 (3.7) | 1,274 (3.7) | 178 (4.6) | 1,096 (3.6) |
| ≥2 | 724 (2.3) | 89 (2.9) | 635 (2.2) | 521 (1.6) | 76 (2.2) | 445 (1.5) | 462 (1.3) | 60 (1.5) | 402 (1.3) |
| ED visits, n (%) |  |  |  |  |  |  |  |  |  |
| 0 | 25,591 (80.4) | 2,512 (80.6) | 23,079 (80.4) | 27,247 (81.6) | 2,743 (80.1) | 24,504 (81.8) | 28,317 (82.3) | 3,165 (81.4) | 25,152 (82.4) |
| 1 | 3,770 (11.8) | 370 (11.9) | 3,400 (11.8) | 3,949 (11.8) | 436 (12.7) | 3,513 (11.7) | 3,897 (11.3) | 462 (11.9) | 3,435 (11.3) |
| ≥2 | 2,463 (7.7) | 233 (7.5) | 2,230 (7.8) | 2,192 (6.6) | 247 (7.2) | 1,945 (6.5) | 2,184 (6.3) | 263 (6.8) | 1,921 (6.3) |

Abbreviations: BMI, body mass index; CCI, Charlson comorbidity index; ED, emergency department; QIVc, cell-based quadrivalent influenza vaccine; QIVe, egg-based quadrivalent influenza vaccine; SD, standard deviation.

^a^Test type was included as a descriptive characteristic and to support the test sensitivity and specific sensitivity analysis. Test type was not assessed for inclusion in model weighting or adjustment.

Supplementary Table 7. Study population selection in each season

| **Selection criterion** | **2017–2018**  **n (%)** | **2018–2019**  **n (%)** | **2019–2020**  **n (%)** |
| --- | --- | --- | --- |
| Individual received QIVc or QIVe during vaccination intake timeframe | 12,740,284 (100) | 13,925,114 (100) | 14,180,727 (100) |
| Individual is aged 4–64 years at time of immunization | 10,277,110 (80.7) | 11,529,852 (82.8) | 11,832,244 (83.4) |
| Individual has any ARFI during influenza season | 3,717,295 (36.2) | 3,944,199 (34.2) | 3,439,970 (29.1) |
| Individual has valid influenza test result within ±7 days of any ARFI date | 54,235 (1.5) | 51,799 (1.3) | 54,752 (1.6) |
| Individual has valid test result >14 days after vaccination | 45,343 (83.6) | 44,708 (86.3) | 44,353 (81.0) |
| Individual meets criteria for number of influenza vaccinations^a^ | 37,351 (82.4) | 38,418 (85.9) | 39,184 (88.3) |
| Individual did not receive any type of influenza vaccine between end of the previous influenza season and start of current season’s vaccination intake timeframe | 37,282 (99.8) | 38,355 (99.8) | 39,098 (99.8) |
| Individual has a transcript record in the Veradigm EHR ≥1 year prior to vaccination date | 33,541 (90.0) | 34,621 (90.3) | 35,482 (90.8) |
| Individual has activity in Komodo claims ≥1 year prior to vaccination date | 31,868 (95.0) | 33,413 (96.5) | 34,422 (97.0) |
| Individual does not have missing data in the EHR fields for sex and geographic region | 31,824 (99.9) | 33,388 (99.9) | 34,398 (99.9) |
| **Full Study Population** | **n=31,824** | **n=33,388** | **n=34,398** |
| QIVc | 3,115 (9.8) | 3,426 (10.3) | 3,890 (11.3) |
| Cases | 726 (23.3) | 723 (21.1) | 979 (25.2) |
| Controls | 2,389 (76.7) | 2,703 (78.9) | 2,911 (74.8) |
| QIVe | 28,709 (90.2) | 29,962 (89.7) | 30,508 (88.7) |
| Cases | 8,390 (29.2) | 9,280 (31.0) | 9,987 (32.7) |
| Controls | 20,319 (70.8) | 20,682 (69.0) | 20,521 (67.3) |

Abbreviations: ARFI, acute respiratory or febrile illness; EHR, electronic health record; QIVc, cell-based quadrivalent influenza vaccine; QIVe, egg-based quadrivalent influenza vaccine.

^a^ Age ≥9 years: does not have >1 influenza immunization between start date of vaccination intake timeframe and test date; age <9 years: does not have (a) >2 influenza vaccine administrations or (b) 2 different vaccine administrations between start date of the vaccination intake timeframe and test date.

Supplementary Figure 1. Proportions of test-positive influenza case and control study populations with at least 1 high-risk medical condition within each vaccine group during each season

QIVc, cell-based quadrivalent influenza vaccine; QIVe, egg-based quadrivalent influenza vaccine.


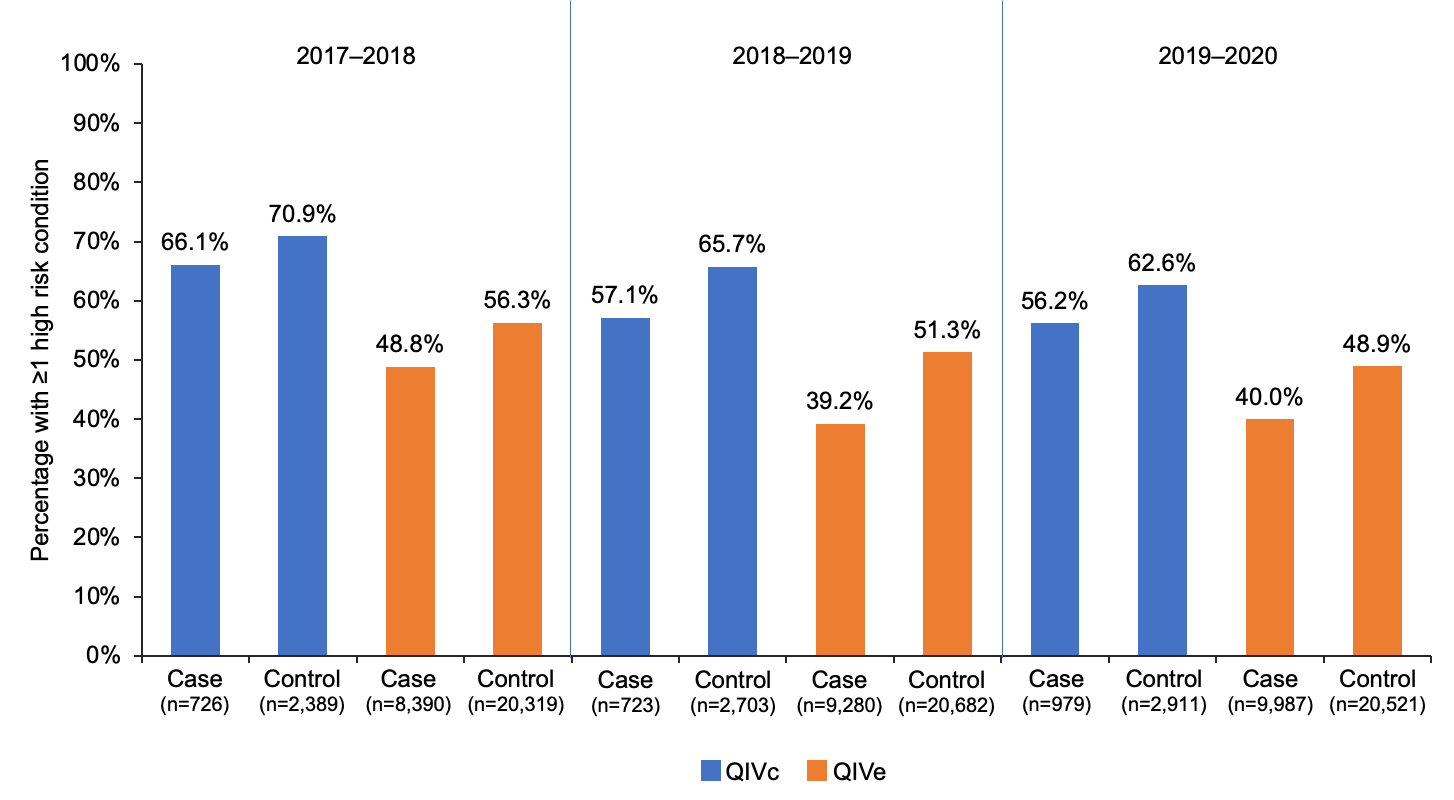


Supplementary Table 8. Demographic characteristics of the study population at index date by case and control populations within each vaccine group

| **Characteristic** | **2017–2018** | | | | **2018–2019** | | | | **2019–2020** | | | |
| --- | --- | --- | --- | --- | --- | --- | --- | --- | --- | --- | --- | --- |
|  | **QIVc** | | **QIVe** | | **QIVc** | | **QIVe** | | **QIVc** | | **QIVe** | |
|  | **Case (n=726)** | **Control (n=2,389)** | **Case (n=8,390)** | **Control (n=20,319)** | **Case (n=723)** | **Control (n=2,703)** | **Case (n=9,280)** | **Control (n=20,682)** | **Case (n=979)** | **Control (n=2,911)** | **Case (n=9,987)** | **Control (n=20,521)** |
| Age, mean (SD) | 41.6 (19.3) | 42.8 (17.1) | 24.2 (21.3) | 29.1 (21.3) | 33.4 (20.7) | 40.3 (17.6) | 17.6 (17.4) | 25.8 (20.4) | 33.6 (19.2) | 38.4 (18.2%) | 17.2 (17.0%) | 24.1 (19.6%) |
| Age group, n (%) |  |  |  |  |  |  |  |  |  |  |  |  |
| 4-17 years | 143 (19.7) | 293 (12.3) | 5,081 (60.6) | 9,483 (46.7) | 263 (36.4) | 437 (16.2) | 6,982 (75.2) | 10,929 (52.8) | 299 (30.5) | 566 (19.4%) | 7,457 (74.7%) | 11,581 (56.4%) |
| 18-49 years | 237 (32.6) | 1,020 (42.7) | 1,464 (17.4) | 5,445 (26.8) | 231 (32.0) | 1,205 (44.6) | 1,292 (13.9) | 5,548 (26.8) | 416 (42.5) | 1,319 (45.3%) | 1,537 (15.4%) | 5,370 (26.2%) |
| 50-64 years | 346 (47.7) | 1,076 (45.0) | 1,845 (22.0) | 5,391 (26.5) | 229 (31.7) | 1,061 (39.3) | 1,006 (10.8) | 4,205 (20.3) | 264 (27.0) | 1,026 (35.2%) | 993 (9.9%) | 3,570 (17.4%) |
| Sex, n (%) |  |  |  |  |  |  |  |  |  |  |  |  |
| Female | 434 (59.8) | 1,611 (67.4) | 4,615 (55.0) | 12,420 (61.1) | 454 (62.8) | 1,774 (65.6) | 5,000 (53.9) | 12,403 (60.0) | 587 (60.0) | 1,934 (66.4%) | 5,354 (53.6%) | 12,244 (59.7%) |
| Male | 292 (40.2) | 778 (32.6) | 3,775 (45.0) | 7,899 (38.9) | 269 (37.2) | 929 (34.4) | 4,280 (46.1) | 8,279 (40.0) | 392 (40.0) | 977 (33.6%) | 4,633 (46.4%) | 8,277 (40.3%) |
| Race, n (%) |  |  |  |  |  |  |  |  |  |  |  |  |
| White | 534 (73.6) | 1,723 (72.1) | 5,586 (66.6) | 13,829 (68.1) | 512 (70.8) | 1,919 (71.0) | 6,211 (66.9) | 14,055 (68.0) | 684 (69.9) | 1,992 (68.4%) | 6,833 (68.4%) | 14,069 (68.6%) |
| Black | 50 (6.9) | 157 (6.6) | 542 (6.5) | 1,344 (6.6) | 34 (4.7) | 137 (5.1) | 600 (6.5) | 1,429 (6.9) | 47 (4.8) | 139 (4.8%) | 467 (4.7%) | 1,260 (6.1%) |
| Asian | 13 (1.8) | 38 (1.6) | 216 (2.6) | 367 (1.8) | 14 (1.9) | 74 (2.7) | 248 (2.7) | 398 (1.9) | 45 (4.6) | 66 (2.3%) | 268 (2.7%) | 354 (1.7%) |
| Other | 6 (0.8) | 24 (1.0) | 240 (2.9) | 459 (2.3) | 12 (1.7) | 29 (1.1) | 280 (3.0) | 495 (2.4) | 17 (1.7) | 47 (1.6%) | 266 (2.7%) | 509 (2.5%) |
| Unknown/not reported | 123 (16.9) | 447 (18.7) | 1,806 (21.5) | 4,320 (21.3) | 151 (20.9) | 544 (20.1) | 1,941 (20.9) | 4,305 (20.8) | 186 (19.0) | 667 (22.9%) | 2,153 (21.6%) | 4,329 (21.1%) |
| Ethnicity, n (%) |  |  |  |  |  |  |  |  |  |  |  |  |
| Hispanic | 42 (5.8) | 106 (4.4) | 731 (8.7) | 1,343 (6.6) | 66 (9.1) | 151 (5.6) | 1,017 (11.0) | 1,692 (8.2) | 102 (10.4) | 181 (6.2%) | 1,057 (10.6%) | 1,592 (7.8%) |
| Non-Hispanic | 643 (88.6) | 2,114 (88.5) | 6,991 (83.3) | 17,194 (84.6) | 572 (79.1) | 2,299 (85.1) | 7,438 (80.2) | 17,132 (82.8) | 767 (78.3) | 2,422 (83.2%) | 7,959 (79.7%) | 16,843 (82.1%) |
| Unknown/not reported | 41 (5.6) | 169 (7.1) | 668 (8.0) | 1,782 (8.8) | 85 (11.8) | 253 (9.4) | 825 (8.9) | 1,858 (9.0) | 110 (11.2) | 308 (10.6%) | 971 (9.7%) | 2,086 (10.2%) |
| Geographic region, n (%) |  |  |  |  |  |  |  |  |  |  |  |  |
| Northeast | 44 (6.1) | 135 (5.7) | 1,152 (13.7) | 1,953 (9.6) | 51 (7.1) | 159 (5.9) | 1,282 (13.8) | 2,090 (10.1) | 66 (6.7) | 203 (7.0%) | 2,142 (21.4%) | 2,744 (13.4%) |
| Midwest | 101 (13.9) | 312 (13.1) | 2,517 (30.0) | 6,725 (33.1) | 58 (8.0) | 200 (7.4) | 1,830 (19.7) | 4,583 (22.2) | 120 (12.3) | 287 (9.9%) | 2,111 (21.1%) | 4,463 (21.7%) |
| South | 550 (75.8) | 1,876 (78.5) | 4,410 (52.6) | 10,908 (53.7) | 552 (76.3) | 2,206 (81.6) | 5,601 (60.4) | 12,977 (62.7) | 652 (66.6) | 2,131 (73.2%) | 5,131 (51.4%) | 12,202 (59.5%) |
| West | 31 (4.3) | 66 (2.8) | 311 (3.7) | 733 (3.6) | 62 (8.6) | 138 (5.1) | 567 (6.1) | 1,032 (5.0) | 141 (14.4) | 290 (10.0%) | 603 (6.0%) | 1,112 (5.4%) |
| Test type, n (%)^a^ |  |  |  |  |  |  |  |  |  |  |  |  |
| Antigen | 700 (96.4) | 2,292 (95.9) | 7740 (92.3) | 18,887 (93.0) | 660 (91.3) | 2,435 (90.1) | 8,653 (93.2) | 19,114 (92.4) | 763 (77.9) | 2,330 (80.0) | 7,902 (79.1) | 16,960 (82.6) |
| Molecular | 21 (2.9) | 91 (3.8) | 517 (6.2) | 1,172 (5.8) | 58 (8.0) | 256 (9.5) | 461 (5.0) | 1,263 (6.1) | 170 (17.4) | 507 (17.4) | 2,007 (20.1) | 3,411 (16.6) |
| Culture | 3 (0.4) | 2 (0.1) | 87 (1.0) | 172 (0.8) | 1 (0.1) | 6 (0.2) | 86 (0.9) | 200 (1.0) | 0 (0.0) | 2 (0.1) | 22 (0.2) | 46 (0.2) |
| Antibody | 2 (0.3) | 4 (0.2) | 46 (0.5) | 88 (0.4) | 4 (0.6) | 6 (0.2) | 80 (0.9) | 105 (0.5) | 46 (4.7) | 72 (2.5) | 56 (0.6) | 104 (0.5) |
| CCI, mean (SD) | 0.5 (1.0) | 0.7 (1.2) | 0.4 (0.9) | 0.5 (1.1) | 0.4 (0.9) | 0.6 (1.2) | 0.3 (0.7) | 0.4 (1.0) | 0.4 (0.9) | 0.5 (1.1) | 0.2 (0.6) | 0.4 (0.9) |
| High-risk conditions, n (%) |  |  |  |  |  |  |  |  |  |  |  |  |
| ≥1 High-risk condition | 480 (66.1) | 1,694 (70.9) | 4,096 (48.8) | 11,449 (56.3) | 413 (57.1) | 1,777 (65.7) | 3,641 (39.2) | 10,620 (51.3) | 550 (56.2) | 1,821 (62.6) | 3,990 (40.0) | 10,042 (48.9) |
| Heart disease and related conditions | 298 (41.0) | 1,065 (44.6) | 1,810 (21.6) | 5,753 (28.3) | 207 (28.6) | 1,046 (38.7) | 1,117 (12.0) | 4,725 (22.8) | 264 (27.0) | 1,002 (34.4) | 1,159 (11.6) | 4,095 (20.0) |
| Metabolic disorders | 287 (39.5) | 1,028 (43.0) | 1,611 (19.2) | 5,206 (25.6) | 209 (28.9) | 982 (36.3) | 1,072 (11.6) | 4,386 (21.2) | 242 (24.7) | 946 (32.5) | 1,139 (11.4) | 3,916 (19.1) |
| Endocrine disorders | 194 (26.7) | 786 (32.9) | 1,274 (15.2) | 4,169 (20.5) | 161 (22.3) | 798 (29.5) | 826 (8.9) | 3,429 (16.6) | 203 (20.7) | 732 (25.1) | 844 (8.5) | 3,085 (15.0) |
| Asthma | 88 (12.1) | 365 (15.3) | 1,449 (17.3) | 3,538 (17.4) | 112 (15.5) | 404 (14.9) | 1,514 (16.3) | 3,401 (16.4) | 150 (15.3) | 440 (15.1) | 1,572 (15.7) | 3,412 (16.6) |
| Blood disorders | 84 (11.6) | 368 (15.4) | 654 (7.8) | 2,126 (10.5) | 70 (9.7) | 402 (14.9) | 508 (5.5) | 1,913 (9.2) | 97 (9.9) | 372 (12.8) | 560 (5.6) | 1,784 (8.7) |
| Obesity (BMI ≥40 kg/m^2^) | 71 (9.8) | 291 (12.2) | 450 (5.4) | 1,844 (9.1) | 55 (7.6) | 299 (11.1) | 326 (3.5) | 1,647 (8.0) | 77 (7.9) | 303 (10.4) | 350 (3.5) | 1,537 (7.5) |
| Weakened immune system | 56 (7.7) | 177 (7.4) | 316 (3.8) | 1,080 (5.3) | 32 (4.4) | 190 (7.0) | 222 (2.4) | 897 (4.3) | 44 (4.5) | 197 (6.8) | 227 (2.3) | 844 (4.1) |
| Neurologic and neurodevelopmental conditions | 10 (1.4) | 32 (1.3) | 475 (5.7) | 956 (4.7) | 15 (2.1) | 34 (1.3) | 605 (6.5) | 1,125 (5.4) | 27 (2.8) | 67 (2.3) | 758 (7.6) | 1,216 (5.9) |
| Chronic lung disease | 32 (4.4) | 152 (6.4) | 238 (2.8) | 1,025 (5.0) | 21 (2.9) | 165 (6.1) | 146 (1.6) | 755 (3.7) | 32 (3.3) | 151 (5.2) | 105 (1.1) | 665 (3.2) |
| Liver disorders | 31 (4.3) | 119 (5.0) | 173 (2.1) | 664 (3.3) | 24 (3.3) | 145 (5.4) | 104 (1.1) | 545 (2.6) | 30 (3.1) | 126 (4.3) | 116 (1.2) | 502 (2.4) |
| Kidney diseases | 24 (3.3) | 91 (3.8) | 135 (1.6) | 487 (2.4) | 15 (2.1) | 72 (2.7) | 59 (0.6) | 330 (1.6) | 17 (1.7) | 64 (2.2) | 74 (0.7) | 286 (1.4) |
| Stroke | 8 (1.1) | 63 (2.6) | 59 (0.7) | 287 (1.4) | 10 (1.4) | 45 (1.7) | 39 (0.4) | 227 (1.1) | 3 (0.3) | 47 (1.6) | 45 (0.5) | 186 (0.9) |
| Baseline all-cause healthcare resource utilization |  |  |  |  |  |  |  |  |  |  |  |  |
| OP visits, mean (SD) | 4.5 (4.7) | 5.5 (6.2) | 5.1 (5.0) | 5.9 (5.7) | 4.6 (4.9) | 5.5 (5.6) | 4.6 (4.4) | 5.7 (5.4) | 4.9 (5.9) | 5.8 (5.7) | 4.6 (4.4) | 5.6 (5.5) |
| IP admissions, n (%) |  |  |  |  |  |  |  |  |  |  |  |  |
| 0 | 685 (94.4) | 2,173 (91.0) | 7,992 (95.3) | 18,910 (93.1) | 691 (95.6) | 2,492 (92.2) | 8,977 (96.7) | 19,428 (93.9) | 930 (95.0) | 2,722 (93.5) | 9,625 (96.4) | 19,385 (94.5) |
| 1 | 29 (4.0) | 139 (5.8) | 257 (3.1) | 915 (4.5) | 24 (3.3) | 143 (5.3) | 229 (2.5) | 883 (4.3) | 36 (3.7) | 142 (4.9) | 263 (2.6) | 833 (4.1) |
| ≥2 | 12 (1.7) | 77 (3.2) | 141 (1.7) | 494 (2.4) | 8 (1.1) | 68 (2.5) | 74 (0.8) | 371 (1.8) | 13 (1.3) | 47 (1.6) | 99 (1.0) | 303 (1.5) |
| ED visits, n (%) |  |  |  |  |  |  |  |  |  |  |  |  |
| 0 | 619 (85.3) | 1,893 (79.2) | 6,999 (83.4) | 16,080 (79.1) | 614 (84.9) | 2,129 (78.8) | 7,939 (85.5) | 16,565 (80.1) | 821 (83.9) | 2,344 (80.5) | 8,545 (85.6) | 16,607 (80.9) |
| 1 | 67 (9.2) | 303 (12.7) | 893 (10.6) | 2,507 (12.3) | 76 (10.5) | 360 (13.3) | 922 (9.9) | 2,591 (12.5) | 105 (10.7) | 357 (12.3) | 1,016 (10.2) | 2,419 (11.8) |
| ≥2 | 40 (5.5) | 193 (8.1) | 498 (5.9) | 1,732 (8.5) | 33 (4.6) | 214 (7.9) | 419 (4.5) | 1,526 (7.4) | 53 (5.4) | 210 (7.2) | 426 (4.3) | 1,495 (7.3) |

^a^Test type was included as a descriptive characteristic and to support the test sensitivity and specific sensitivity analysis. Test type was not assessed for inclusion in model weighting or adjustment.

Abbreviations: BMI, body mass index; CCI, Charlson comorbidity index; ED, emergency department; QIVc, cell-based quadrivalent influenza vaccine; QIVe, egg-based quadrivalent influenza vaccine; SD, standard deviation.

Supplementary Table 9. Primary analyses: rVEs of QIVc versus QIVe against positive influenza test result of the full study population, by season

| Model, rVE (95% CI) | 2017–2018 | 2018–2019 | 2019–2020 |
| --- | --- | --- | --- |
| Cases/controls (n/n) | 9,116/22,708 | 10,003/23,385 | 10,966/23,432 |
| Mode1 1: unadjusted | 26.4% (19.7%–32.5%) | 40.4% (35.1%–45.3%) | 30.9% (25.4%–36.0%) |
| Model 2: IPTW | 17.9% (10.4%–24.7%) | 16.6% (9.5%–23.1%) | 13.6% (6.8%–19.8%) |
| Model 3: multivariable regression | 13.0% (4.8%–20.6%) | 14.1% (5.9%–21.6%) | 3.4% (–4.7% to 11.0%) |
| **Model 4: Main analysis, doubly robust adjusted (IPTW + multivariable regression)** | **14.8% (7.0%–22.0%)** | **12.5% (4.7%–19.6%)** | **10.0% (2.7%–16.7%)** |

Abbreviations: CI, confidence interval; IPTW, inverse probability of treatment weighting; QIVc, cell-based quadrivalent influenza vaccine; QIVe, egg-based quadrivalent influenza vaccine; rVE, relative vaccine effectiveness.

Supplementary Table 10. Sensitivity analyses: rVEs of QIVc versus QIVe against positive influenza test result of the full study population, by season

| Analysis | 2017–2018 | 2018–2019 | 2019–2020 |
| --- | --- | --- | --- |
| **Propensity to be tested** |  |  |  |
| Cases/controls (n/n) | 9,116/22,708 | 10,003/23,385 | 10,966/23,432 |
| Original cases/original controls (%/%) | 100/100 | 100/100 | 100/100 |
| Doubly robust adjusted model, rVE (95% CI) | 14.7% (6.8%–21.9%) | 12.3% (4.5%–19.4%) | 9.8% (2.6%–16.6%) |
| **Peak season** |  |  |  |
| Cases/controls (n/n) | 8,002/17,783 | 9,245/18,439 | 10,563/20,251 |
| Original cases/original controls (%/%) | 87.8/78.3 | 92.4/78.8 | 96.3/86.4 |
| Doubly robust adjusted model, rVE (95% CI) | 14.8% (6.3%–22.6%) | 9.4% (0.8%–17.3%) | 10.8% (3.3%–17.7%) |
| **Matched index week** |  |  |  |
| Cases/controls (n/n) | 9,116/18,232 | 10,003/10,003 | 10,966/10,966 |
| Matching ratio | 1:2 | 1:1 | 1:1 |
| Original cases/original controls (%/%) | 100.0/80.3 | 100.0/42.8 | 100.0/46.8 |
| Doubly robust adjusted model, rVE (95% CI) | 16.4% (8.4%–23.7%) | 12.8% (3.4%–21.2%) | 13.6% (5.4%–21.2%) |
| **Exclusion of antibody and culture tests** | | | |
| Cases/controls (n/n) | 8,978/22,442 | 9,832/23,068 | 10,842/23,208 |
| Original cases/original controls (%/%) | 98.5/98.8 | 98.3/98.6 | 98.9/99.0 |
| Doubly robust adjusted model, rVE (95% CI) | 16.2% (8.5%–23.3%) | 13.8% (6.1%–20.9%) | 13.0%  (5.7%–19.8%) |

Abbreviations: CI, confidence interval; IPTW, inverse probability of treatment weighting; QIVc, cell-based quadrivalent influenza vaccine; QIVe, egg-based quadrivalent influenza vaccine; rVE, relative vaccine effectiveness.

Supplementary Table 11. Exploratory multivariable regression sensitivity analysis: rVEs of QIVc versus QIVe against positive influenza test result of the antigen and molecular test population, by season

| Matched analysis | 2017–2018 | 2018–2019 | 2019–2020 |
| --- | --- | --- | --- |
| Cases/controls (n/n) | 8,978/22,442 | 9,832/23,068 | 10,842/23,208 |
| Original cases/original controls (%/%) | 98.5/98.8 | 98.3/98.6 | 98.9/99.0 |
| Model 3 (multivariable regression) limited to antigen and molecular tests, rVE (95% CI) | 13.6% (5.4%–21.1%) | 14.5% (6.3%–22.0%) | 3.7% (–4.6% to 11.4%) |
| Exploratory sensitivity: Simulation accounting for potential misclassification, rVE (95% CI) | 10.8% (5.4%–15.9%) | 11.7% (6.7%–16.8%) | 3.4% (–1.2% to 7.8%) |

Abbreviations: CI, confidence interval; QIVc, cell-based quadrivalent influenza vaccine; QIVe, egg-based quadrivalent influenza vaccine; rVE, relative vaccine effectiveness.

Supplementary Table 12. Post hoc multivariable regression analysis: rVEs of QIVc versus QIVe against positive influenza test result of the matched on week population, by season

| Matched analysis | 2017–2018 | 2018–2019 | 2019–2020 |
| --- | --- | --- | --- |
| **Full season** |  |  |  |
| Cases/controls (n/n) | 9,116/18,232 | 10,003/10,003 | 10,966/10,966 |
| Matching ratio | 1:2 | 1:1 | 1:1 |
| Original cases/original controls (%/%) | 100.0/80.3 | 100.0/42.8 | 100.0/46.8 |
| Multivariable adjusted model, rVE (95% CI) | 14.0% (5.7%–21.7%) | 14.2% (4.8%–22.6%) | 6.5% (–2.5% to 14.8%) |
| **Peak season** |  |  |  |
| Cases/controls (n/n) | 8,002/16,001 | 9,245/9,237 | 10,563/10,563 |
| Matching ratio | 1:2 | 1:1 | 1:1 |
| Original cases/original controls (%/%) | 87.8/70.5 | 92.4/39.5 | 96.3/45.1 |
| Multivariable adjusted model, rVE (95% CI) | 14.5% (5.6% –22.6%) | 13.5% (4.6%–21.5%) | 6.8% (–2.4% to 15.2%) |

Abbreviations: CI, confidence interval; IPTW, inverse probability of treatment weighting; QIVc, cell-based quadrivalent influenza vaccine; QIVe, egg-based quadrivalent influenza vaccine; rVE, relative vaccine effectiveness.

Supplementary Table 13. Influenza virus strains included in QIVc and QIVe over the study period

| Season | Influenza type | WHO prototype recommendation, egg | QIVc CVV | QIVc CVV type |
| --- | --- | --- | --- | --- |
| 2017-2018 | A(H1N1) | A/Michigan/45/2015 | A/Singapore/GP1908/2015 IVR-180 | Egg-derived |
|  | A(H3N2) | A/Hong Kong/4801/2014 | A/Singapore/GP2050/2015 | Cell-derived |
|  | B/Victoria | B/Brisbane/60/2008 | B/Hong Kong/259/2010 | Egg-derived |
|  | B/Yamagata | B/Phuket/3073/2013 | B/Utah/9/2014 | Egg-derived |
| 2018-2019 | A(H1N1) | A/Michigan/45/2015 | A/Singapore/GP1908/2015 IVR-180 | Egg-derived |
|  | A(H3N2) | A/Singapore/INFIMH-16-0019/2016 | A/North Carolina/04/2016 | Cell-derived |
|  | B/Victoria | B/Colorado/06/2017 | B/Iowa/06/2017 | Cell-derived |
|  | B/Yamagata | B/Phuket/3073/2013 | B/Singapore/INFTT-16-0610/2016 | Cell-derived |
| 2019-2020 | A(H1N1) | A/Brisbane/02/2018 | A/Idaho/07/2018 | Cell-derived |
|  | A(H3N2) | A/Kansas/14/2017 | A/Indiana/08/2018 | Cell-derived |
|  | B/Victoria | B/Colorado/06/2017 | B/Iowa/06/2017 | Cell-derived |
|  | B/Yamagata | B/Phuket/3073/2013 | B/Singapore/INFTT-16-0610/2016 | Cell-derived |

Abbreviations: CVV, candidate vaccine virus; QIVc, QIVc, cell-based quadrivalent influenza vaccine; QIVe, egg-based quadrivalent influenza vaccine; WHO, World Health Organization.

References

1. Thompson MG, Kwong JC, Regan AK, et al. Influenza vaccine effectiveness in preventing influenza-associated hospitalizations during pregnancy: a multi-country retrospective test negative design study, 2010–2016. *Clin Infect Dis* 2019; 68(9):1444–1453.

2. Austin PC. An introduction to propensity score methods for reducing the effects of confounding in observational studies. *Multivariate Behav Res* 2011; 46(3):399–424.

3. Greenland S. Basic methods for sensitivity analysis and external adjustment. In: Rothman KJ, Greenland S, eds. *Modern Epidemiology*. 2nd ed. Philadelphia: Lippincott Williams & Wilkins; 1998: 343–357.

4. Vandenbroucke JP, Pearce N. Test-negative designs: differences and commonalities with other case-control studies with "other patient" controls. *Epidemiology* 2019; 30(6):838–844.

5. Chua H, Feng S, Lewnard JA, et al. The use of test-negative controls to monitor vaccine effectiveness: a systematic review of methodology. *Epidemiology* 2020; 31(1):43–64.

6. Hastie T, Tibshirani R, Friedman J. *The Elements of Statistical Learning: Data Mining, Inference, and Prediction* [Internet]. 2nd ed. Springer New York; 2009. https://link.springer.com/book/10.1007/978-0-387-84858-7. Accessed 3 August 2023.

7. Harrell FE Jr. SAS Macro AXISSPEC [Internet]. 1986. Available at: https://biostat.app.vumc.org/wiki/pub/Main/SasMacros/survrisk.txt. Accessed 3 August 2023.

8. Rutherford MJ, Crowther MJ, Lambert PC. The use of restricted cubic splines to approximate complex hazard functions in the analysis of time-to-event data: a simulation study. *Journal of Statistical Computation and Simulation* 2015; 85(4):777–793.

9. Hertzmark E, Li R, Spiegelman D. SAS Macro MAKESPL [Internet]. Available at: https://ysph.yale.edu/cmips/research/software/makespl/. Accessed 3 August 2023.

10. Bang H, Robins JM. Doubly robust estimation in missing data and causal inference models. *Biometrics* 2005; 61(4):962–973.

11. Parikh R, Mathai A, Parikh S, Chandra Sekhar G, Thomas R. Understanding and using sensitivity, specificity and predictive values. *Indian J Ophthalmol* 2008; 56(1):45–50.

12. Trombetta VK, Chan YL, Bankowski MJ. Are rapid influenza antigen tests still clinically useful in today's molecular diagnostics world? *Hawaii J Med Public Health* 2018; 77(9):226–230.

13. Vega T, Lozano JE, Meerhoff T, et al. Influenza surveillance in Europe: establishing epidemic thresholds by the moving epidemic method. *Influenza Other Respir Viruses* 2013; 7(4):546–558.

14. Centers for Disease Control and Prevention. FluView interactive: national, regional, and state level outpatient illness and viral surveillance. Available at: https://gis.cdc.gov/grasp/fluview/fluportaldashboard.html. Accessed 3 August 2023.

15. Malosh RE, McGovern I, Monto AS. Influenza during the 2010–2020 decade in the United States: seasonal outbreaks and vaccine interventions. *Clin Infect Dis* 2023; 76(3):540–549.

16. Boikos C, McGovern I, Ortiz JR, Puig-Barberà J, Versage E, Haag M. Relative vaccine effectiveness of adjuvanted trivalent influenza vaccine over three consecutive influenza seasons in the United States. *Vaccines (Basel)* 2022; 10(9):1456.

17. Centers for Disease Control and Prevention. Rapid influenza diagnostic tests. Updated 25 October 2016. Available at: https://www.cdc.gov/flu/professionals/diagnosis/clinician_guidance_ridt.htm. Accessed 3 August 2023.

18. Fox MP, Lash TL, Greenland S. A method to automate probabilistic sensitivity analyses of misclassified binary variables. *Int J Epidemiol* 2005; 34(6):1370–1376.

19. Lash TL, Fink AK. Semi-automated sensitivity analysis to assess systematic errors in observational data. *Epidemiology* 2003; 14(4):451­–458.

20. Food and Drug Administration. 510(k) Substantial equivalence determination decision summary for Acucy Influenza A&B Test. 2018. Available at: https://www.accessdata.fda.gov/cdrh_docs/reviews/K182001.pdf. Accessed 3 August 2023.

21. Food and Drug Administration. 510(k) Substantial equivalence determination decision summary for SofiaTM Analyzer and Influenza A+B FIA. 2011. Available at: https://www.accessdata.fda.gov/cdrh_docs/reviews/K162438.pdf. Accessed 3 August 2023.

22. Food and Drug Administration. 510(k) Substantial equivalence determination decision summary for BioSign® Flu A+B. 2010. Available at: https://www.accessdata.fda.gov/cdrh_docs/reviews/K083746.pdf. Accessed 3 August 2023.

23. Maignan M, Viglino D, Hablot M, et al. Diagnostic accuracy of a rapid RT-PCR assay for point-of-care detection of influenza A/B virus at emergency department admission: a prospective evaluation during the 2017/2018 influenza season. *PLoS One* 2019; 14(5):e0216308.
